# Transcriptomic Profiling of the Adaptive and Innate Immune Responses of Atlantic Salmon to *Renibacterium salmoninarum* Infection

**DOI:** 10.3389/fimmu.2020.567838

**Published:** 2020-10-28

**Authors:** Khalil Eslamloo, Albert Caballero-Solares, Sabrina M. Inkpen, Mohamed Emam, Surendra Kumar, Camila Bouniot, Ruben Avendaño-Herrera, Eva Jakob, Matthew L. Rise

**Affiliations:** ^1^Department of Ocean Sciences, Memorial University of Newfoundland, St. John's, NL, Canada; ^2^Cargill Innovation Center—Colaco, Calbuco, Chile; ^3^Facultad Ciencias de la Vida, Viña del Mar, and FONDAP Interdisciplinary Center for Aquaculture Research (INCAR), Universidad Andrés Bello, Santiago, Chile

**Keywords:** Bacterial Kidney Disease (BKD), *Salmo salar*, microarray, antibacterial responses, teleost, infection level, transcriptome, individual-dependent immune response to pathogen

## Abstract

Bacterial Kidney Disease (BKD), which is caused by a Gram-positive, intracellular bacterial pathogen (*Renibacterium salmoninarum*), affects salmonids including Atlantic salmon (*Salmo salar*). However, the transcriptome response of Atlantic salmon to BKD remained unknown before the current study. We used a 44K salmonid microarray platform to characterise the global gene expression response of Atlantic salmon to BKD. Fish (~54 g) were injected with a dose of *R. salmoninarum* (H-2 strain, 2 × 10^8^ CFU per fish) or sterile medium (control), and then head kidney samples were collected at 13 days post-infection/injection (dpi). Firstly, infection levels of individuals were determined through quantifying the *R. salmoninarum* level by RNA-based TaqMan qPCR assays. Thereafter, based on the qPCR results for infection level, fish (*n* = 5) that showed no (control), higher (H-BKD), or lower (L-BKD) infection level at 13 dpi were subjected to microarray analyses. We identified 6,766 and 7,729 differentially expressed probes in the H-BKD and L-BKD groups, respectively. There were 357 probes responsive to the infection level (H-BKD vs. L-BKD). Several adaptive and innate immune processes were dysregulated in *R. salmoninarum*-infected Atlantic salmon. Adaptive immune pathways associated with lymphocyte differentiation and activation (e.g., lymphocyte chemotaxis, T-cell activation, and immunoglobulin secretion), as well as antigen-presenting cell functions, were shown to be differentially regulated in response to BKD. The infection level-responsive transcripts were related to several mechanisms such as the JAK-STAT signalling pathway, B-cell differentiation and interleukin-1 responses. Sixty-five microarray-identified transcripts were subjected to qPCR validation, and they showed the same fold-change direction as microarray results. The qPCR-validated transcripts studied herein play putative roles in various immune processes including pathogen recognition (e.g., *tlr5*), antibacterial activity (e.g., *hamp* and *camp*), regulation of immune responses (e.g., *tnfrsf11b* and *socs1*), T-/B-cell differentiation (e.g., *ccl4, irf1* and *ccr5*), T-cell functions (e.g., *rnf144a, il13ra1b* and *tnfrsf6b*), and antigen-presenting cell functions (e.g., *fcgr1*). The present study revealed diverse immune mechanisms dysregulated by *R. salmoninarum* in Atlantic salmon, and enhanced the current understanding of Atlantic salmon response to BKD. The identified biomarker genes can be used for future studies on improving the resistance of Atlantic salmon to BKD.

## Introduction

Due to the limitation of naturally supplied aquatic stocks and a growing human population, fish aquaculture has become one of the main sources fulfilling the global demand for fish consumption (1, 2). However, aquaculture faces several health challenges (e.g., bacterial or viral diseases), and an enhanced understanding of the fish immune and physiological responses to pathogens may help combat epidemics in aquaculture environments. Atlantic salmon (*Salmo salar*) is one of the most economically important fish species prevalently farmed in marine aquaculture worldwide (1, 3), and is susceptible to several Gram-positive and Gram-negative bacterial pathogens causing high mortalities and economic losses (4, 5). *Renibacterium salmoninarum* is a Gram-positive intracellular pathogen that causes Bacterial Kidney Disease (BKD) in Atlantic salmon and other salmonids [e.g., sockeye salmon (*Oncorhynchus nerka*) and rainbow trout (*O. mykiss*)] (6). BKD has been reported in Canada, Chile and several other countries worldwide (6, 7), and can cause up to 40% cumulative mortality in farmed Atlantic salmon (8, 9).

*R. salmoninarum* infection begins in the fish head kidney through formation of granulomas, and then develops in other internal organs (e.g., posterior kidney and liver) of the fish (7–9). Mortalities caused by BKD may be associated with immunosuppressive effects of *R. salmoninarum* on the host (10). An enhanced understanding of the Atlantic salmon response to *R. salmoninarum* can aid in the development of preventive management tools for BKD (e.g., vaccines and therapeutic diets). Previous *in vivo* and *in vitro* studies examined gene expression responses to *R. salmoninarum* in rainbow trout (11) and Chinook salmon (*O. tshawytscha*) (12) kidney as well as rainbow trout macrophages (13) and Atlantic Salmon Kidney (ASK) cell line (14). A genomics-based study used Suppression Subtractive Hybridisation (SSH) to identify BKD-responsive genes in Chinook salmon (15). A recent study reported expression responses of 22 genes (e.g., transcripts encoding interleukins and interferons) in the head kidney of Atlantic salmon infected with *R. salmoninarum* at different temperatures (16). Since vaccination with formalin-killed *R. salmoninarum* was found to enhance the resistance of salmonids to BKD (9), microarrays were previously used to profile the transcriptome response of Atlantic salmon to formalin-killed *R. salmoninarum* bacterin (17). Although this previous study enhanced our understanding of the Atlantic salmon immune response to *R. salmoninarum*-derived antigens, the transcriptome response and molecular pathways underlying Atlantic salmon response to live *R. salmoninarum* pathogen remained uncharacterised before the current study. Considering the immunomodulatory effects of *R. salmoninarum* on its host, profiling the BKD-responsive genes in Atlantic salmon is of prominent importance for the development of methods for combating BKD.

Microarray analyses can determine the transcriptome profile of immunological responses in a species (18). In addition to the aforementioned *R. salmoninarum* bacterin study (17), microarrays were previously employed to profile the antibacterial responses of Atlantic salmon to other bacterial pathogens such as *Piscirickettsia salmonis* (19), and *Aeromonas salmonicida* (20, 21) as well as commercial vaccines (e.g., for immunisation against *Yersinia ruckeri* and *Vibrio* spp.) (22, 23).

The consortium for Genomic Research on All Salmonids Project (cGRASP)-designed Agilent 44K salmonid oligonucleotide microarray (24) was previously used in several immune-related studies in Atlantic salmon and rainbow trout (17, 25–29). In the present study, we used this powerful microarray platform to identify Atlantic salmon head kidney transcripts responsive to *R. salmoninarum* pathogen and determine if the level of *R. salmoninarum* infection [i.e., higher and lower susceptibility levels corresponding to higher and lower infection levels, respectively, at 13 days post-infection/injection (dpi) as determined by TaqMan assays] influenced the Atlantic salmon response to BKD. All the fish in the present study received the same dose of *R. salmoninarum*, but individuals with various levels of infection (i.e., higher and lower infection level groups) at 13 dpi, as shown by reverse transcription—quantitative polymerase chain reaction (qPCR) TaqMan assays, were used for transcriptome analyses. Complementary to our previous investigation (17), the present study identified the genes and molecular pathways associated with Atlantic salmon response to *R. salmoninarum* pathogen, and provided a set of valuable biomarkers for future BKD-related investigations. Furthermore, the infection level-responsive genes identified herein broaden horizons for the understanding of the correlations between *R. salmoninarum* level and Atlantic salmon antibacterial responses.

## Materials and Methods

### Animals

Atlantic salmon parr [54 ± 6 g; (mean ± SE)] were purchased from a local salmon production hatchery and transferred to the Cargill Innovation Center—Colaco, Chile. Before transportation and by sanitary regulations, qPCR assays were used to monitor Atlantic salmon diseases, and fish were certified to be free of pathogens previously reported in Chilean salmon farms [i.e., infectious salmon anaemia virus (ISAV), infectious pancreatic necrosis virus (IPNV), *P. salmonis* and *R. salmoninarum*; (30)]. Fish were distributed to seven circular tanks (200 L tanks; 68 fish per tank), using a freshwater [i.e., five practical salinity unit (psu)] flow-through system (4.3 L min^−1^). Prior to the infection trial, fish were acclimatised to the experimental conditions for 2 weeks and held at 10–11°C water temperature under a 24 h light photoperiod. Fish were fed to satiation using a standard EWOS commercial diet. Water quality parameters were monitored daily (i.e., temperature, oxygen saturation, salinity, and pH). Fish were fasted 12 h before all experimental procedures (e.g., injection and sampling), and were anaesthetised using Benzocaine (150 μl L^−1^ BZ-20®, Veterquímica S.A., Maipú, Santiago, Chile) before handlings and injections. All procedures in this study were conducted following the guidelines of the Canadian Council on Animal Care (31).

### *R. salmoninarum* Strain and Culture

The previously characterised Chilean strain of *R. salmoninarum* (H-2), obtained from cage-cultured Atlantic salmon in 2014, was used for the present study. This strain was isolated from fish with clinical signs of BKD in southern Chile. Previous studies showed that strain H-2 has high siderophore production, which can result in high virulence potential (14, 32, 33). The strain identification was confirmed as *R. salmoninarum* with nested PCR, as described by Chase and Pascho (34), and culture purity was confirmed by Gram-staining, cell morphology and colony morphology (33). Stock cultures were maintained frozen at −80°C in Cryobille tubes (AES Laboratoire, Combourg, France) or in KDM-2 with 15% glycerol. The bacteria were cultured in KDM-2 [1% tryptone (AES Laboratoire), 0.05% yeast extract (AES Laboratoire), 0.1% L-cysteine hydrochloride (US Biological, Salem, MA), 10% fetal bovine serum (Biological Industries, Cromwell, CT); Evelyn 1977] agar under aerobic conditions for 10–15 days at 15°C, and with not more than two subcultures grown from glycerol-amended stock cultures.

### Pathogen Infection

For BKD challenge, inocula were prepared through collecting the bacterial cells from KDM-2 plates and re-suspending in KDM-2 broth (4 ml). After reaching the logarithmic phase, bacterial culture was re-inoculated in KDM-2 broth (400 ml) at 15°C with agitation (50 rpm) to achieve an initial bacterial concentration of 3 × 10^9^ cells ml^−1^, which was determined using direct microscopy count. All fish in the BKD treatment (i.e., five tanks) were intraperitoneally injected with 200 μl of *R. salmoninarum* to obtain a final dose of 2 × 10^8^ colony-forming units (CFU) per fish, as determined by the direct plate count. Fish were challenged using a single high dose of *R. salmoninarum*, as we aimed to study Atlantic salmon response to a lethal level of this pathogen. There was no previous study on mortalities of Atlantic salmon challenged with *R. salmoninarum*, strain H-2. However, a previous study reported fast and high mortalities (i.e., 100% mortality within 15 days) of Atlantic salmon (i.e., 50–70 g) challenged with 10^8^ cells of other strains of *R. salmoninarum* (35). Therefore, we selected a *R. salmoninarum* dose slightly higher than that used in Daly et al. (35) to ensure high mortalities and a strong immune response of Atlantic salmon to BKD. Three tanks in the BKD group were used for monitoring fish mortality, and 2 tanks were used for sampling. Fish in the control group (i.e., two tanks) were injected with 200 μl of sterile KDM-2 broth. Fish in both treatments were fed as described above and held in optimal conditions (i.e., temperature 10–11°C and oxygen saturation above 90%) during the infection trial. Mortalities were recorded daily. Mortalities started at 24 dpi and 100% mortality was seen at 38 dpi. Supplementary Figure S1 shows the cumulative mortality in each tank. Considering mortality data and the objectives of the present study, we selected 13 dpi for sampling and transcriptome analyses. Our sampling time point (i.e., 13 dpi) was approximately at the mid-point of the infection challenge between the start of the infection and the onset of mortalities; we anticipated that it would provide understanding of both early and late immune responses to *R. salmoninarum*. Further, with respect to chronic development of BKD, 13 dpi was considered as the adequate time for pathogen accumulation and for the *R. salmoninarum*-infected fish to show the individual-dependent variations in immune response; correspondingly, it was a suitable time point for studying the differences between the response of fish with higher and lower detected infection levels (i.e., higher and lower susceptible individuals). All the fish in the current study were infected with the same dose of *R. salmoninarum*, and infection level represents the quantitative results of pathogen detection, determined by Taq-Man qPCR assays for each individual at 13 dpi.

### Sampling and RNA Extraction

Ten fish in each experimental tank (i.e., two tanks per treatment; *n* = 20), were euthanized using an overdose of Benzocaine [i.e., initial anaesthesia using 150 μl L^−1^ BZ-20® followed by euthanasia using 300 μl L^−1^ BZ-20® (Veterquímica S.A.)] at 13 dpi. Thereafter, individuals were dissected, and head kidney samples were collected and stored in RNA*later* (Thermo Fisher Scientific, Waltham, MA) at 4°C for 24 h. Then, RNA*later* was removed, and samples were kept at −80°C until RNA extraction. Supplementary Figure S2 illustrates the overall experimental design of the present study. The average (mean ± SE) fish weight was 56.7 ± 1.7 g and 50.96 ± 1.6 g for all sampled fish (*n* = 20) in the control and BKD group, respectively, at 13 dpi.

Total RNA was extracted using TRIzol® Reagent (Thermo Fisher Scientific) following the manufacturer's instructions. Head kidney samples (50–100 mg) were TRIzol-lysed using a tissue homogeniser (Precellys 24, Bertin Instruments, Montigny-Le-Bretonneux, France) before total RNA extraction. To remove residual genomic DNA and enhance RNA quality, total RNA samples were on-column DNase-treated and column-purified using PureLink^TM^ RNA Mini Kit (Thermo Fisher Scientific) and PureLink^TM^ DNAse set (Thermo Fisher Scientific), according to the manufacturer's protocol.

The column-purified RNAs were quantified using a NanoDrop spectrophotometer (ND-1000), and RNA integrity was assessed using 1% agarose gel electrophoresis. The RNA samples used in the microarray and qPCR analyses of the current study showed high integrity (i.e., tight 18S and 28S ribosomal RNA bands) and purity (i.e., A260/230 > 1.7 and A260/280 ratios > 1.8).

### TaqMan Assays for Infection Level Detection and Sample Selection

The reverse transcription—quantitative polymerase chain reaction is referred to as qPCR in the current study. TaqMan qPCR assays were used to assess the infection level in DNase-treated and column-purified RNA samples of Atlantic salmon from both BKD and control groups at 13 dpi. We aimed to test if individuals that were injected with the same dose of live *R. salmoninarum* showed different infection levels at 13 dpi. TaqMan primers and probe for *R. salmoninarum 16S ribosomal RNA* (in-house developed by Cargill Innovation) were used for BKD detection, and Atlantic salmon *elongation factor 1 alpha-1* (*ef1a1*) (36) was used as an internal control. Supplementary Table S1 shows the sequence and quality control results of TaqMan primers and probes used in the current study. All TaqMan assays in the present study were conducted in duplicate using the ViiA 7 Real-Time PCR system (384-well format) (Applied Biosystems, Thermo Fisher Scientific) and AgPath-ID One-Step RT-PCR Reagents (Applied Biosystems, Thermo Fisher Scientific). The assays were performed using 13 μl reactions consisting of 6.5 μl 2X RT-PCR Buffer, 0.52 μl 25X RT-PCR Enzyme Mix, 0.88 μl Detection Enhancer, 0.39 μl (600 nM) forward primer, 0.59 μl (900 nM) reverse primer, 0.23 μl (175 nM) Probe, 0.89 μl DEPC-treated water and 3 μl RNA template (50–100 ng, see below). The TaqMan PCR program comprised one cycle of 45°C for 10 min (reverse transcription), one cycle of 95°C for 10 min, and 45 cycles of 95°C for 15 s and 60°C for 45 s. A pool consisting of equal amounts of RNA from five individuals in the BKD group was used as a template to test the performance and amplification efficiencies of primers. Primer quality control tests were performed using a 5-point, 3-fold serial dilution of the pool RNA template, starting with 100 ng of input total RNA per reaction and a no-template control. Then, the infection level assays for each sample were measured using 50 ng of RNA input in each TaqMan reaction. Also, no-template, positive (i.e., a pool of RNA from 10 fish in BKD group) and negative (i.e., DNase-treated and column-purified RNA from the skin of a non-infected Atlantic salmon) controls were included in all TaqMan assays (i.e., both *R. salmoninarum 16S ribosomal RNA* and Atlantic salmon *ef1a1*). Using QuantStudio™ Real-Time PCR Software (Version 1. 3) (Applied Biosystems, Thermo Fisher Scientific), the fluorescence threshold cycle (C_T_) values and amplification efficiencies of primers were used to calculate the relative quantity (RQ; i.e., calibrated to the sample with the lowest normalised expression level) of *R. salmoninarum 16S ribosomal RNA* through normalisation to Atlantic salmon *ef1a1* (C_T_ range: 18.03–18.81). Five head kidney samples from the control group were included in the microarray and qPCR studies. In addition, five fish with *16S ribosomal RNA* C_T_ values above 25 were selected as samples with lower infection level at 13 dpi or lower susceptibility (L-BKD) for microarray and qPCR analyses (Supplementary Figure S3), and five fish showing C_T_ values below 22 were selected as samples with higher infection level at 13 dpi or higher susceptibility (H-BKD) and were included in the microarray and qPCR studies (Supplementary Figure S3). The level of *R. salmoninarum* infection, which can also reflect the susceptibility, at 13 dpi significantly varied between the H-BKD and L-BKD groups (Supplementary Figure S3); therefore, these samples were used to test if various levels of infection at 13 dpi can influence the transcript expression response of Atlantic salmon to *R. salmoninarum*. Moreover, there was no significant correlation (*p* = 0.6) between weight and the infection level of individuals at 13 dpi, and no significant difference among the weight of individuals in the control, H-BKD and L-BKD groups (*n* = 5). Correspondingly, the differences seen between the infection level of fish [i.e., individuals with higher (H-BKD) and lower (L-BKD) infection level, as detected by Taq-Man assays] at 13 dpi can be associated with the individual-dependent variations in immune response and susceptibility to BKD.

### Microarray Experimental Design and Hybridisation

The selected head kidney samples of five individuals in the control group, five individuals showing higher level of *R. salmoninarum* infection (H-BKD) at 13 dpi and five individuals with lower level of *R. salmoninarum* infection (L-BKD) at 13 dpi were subjected to microarray analysis (i.e., 15 samples in total). The current microarray experiment was designed based upon the Minimum Information About a Microarray Experiment (MIAME) guidelines (37), and it was conducted using cGRASP-designed Agilent 44K salmonid oligonucleotide microarrays (24). Briefly, anti-sense amplified RNA (aRNA) for each sample was *in vitro* transcribed using 1 μg of DNase-treated and column-purified RNA and the Amino Allyl MessageAmp™ II aRNA Amplification Kit (Ambion, Thermo Fisher Scientific), following the manufacturer's instructions. Gel electrophoresis and NanoDrop spectrophotometry were utilised to quality-check and measure the concentration, respectively, of aRNAs. The common reference was composed of an aRNA pool of all 15 samples (i.e., 15 μg from each sample) in the study. Twenty micrograms of aRNA from each sample or common reference were precipitated through a standard ethanol precipitation method and re-suspended in coupling buffer (Ambion, Thermo Fisher Scientific). Thereafter, the experimental samples were labelled with Cy5 (GE Healthcare Life Sciences, Buckinghamshire, UK), whereas the common reference was labelled with Cy3 (GE Healthcare Life Sciences), according to the manufacturer's instructions. Labelling efficiency and labelled aRNA concentrations were assessed using NanoDrop spectrophotometry (i.e., the microarray feature). For each individual sample, 825 ng of its corresponding Cy5-labelled aRNA and 825 ng of Cy3-labelled common reference were pooled, fragmented and co-hybridised to a 44K microarray following the manufacturer's recommendation (Agilent, Santa Clara, CA). The hybridisation of arrays was performed at 65°C for 17 h with rotation (10 rpm) using an Agilent hybridisation oven. According to the manufacturer' instruction, the wash buffers were supplemented with 10% Triton X-102 (Agilent) at the concentration of 0.5 μl ml^−1^. Slides were washed with Gene Expression Wash Buffer 1 (Agilent) and then Gene Expression Wash Buffer 2 (Agilent), using 50 ml Conical Centrifuge Tubes and a rocker platform [VWR Rocker (Radnor, PA); speed 40, tilt 6], for 5 min at room temperature. Slides were dried by centrifuging at 200 × *g* for 5 min at room temperature prior to scanning.

### Microarray Data Acquisition and Analyses

Microarray slides were scanned at 5 μm resolution using a SureScan Microarray Scanner System (Agilent) and Microarray Scan Control Software v.9.1 following the Agilent HD 2-color gene expression microarray scan protocol. The signal intensity data were extracted and Loess-normalised using Agilent Feature Extraction Software v12.0 (Agilent). In GeneSpring Software v14.9 (Agilent), probes of low or marginal quality as well as absent values in more than 25% of all 15 arrays were removed from the dataset, and the missing values were imputed. The final dataset, passing the quality control in GeneSpring and subjected to the statistical analyses, consisted of 33,780 probes for all arrays (GEO accession number: GSE150335). The statistical analyses of microarray data were conducted using GeneSpring Software v14.9. One-way ANOVA was used to determine if there were any significant differences among groups (*p* ≤ 0.01). This analysis was followed by Tukey's multiple comparisons *post-hoc* test to identify significant differences (*p* ≤ 0.01) between groups, and Multiple Testing Correction was performed using the Benjamini-Hochberg procedure.

The differentially expressed probes (DEPs) were re-annotated using the contigs (24) based on which the 60-mer oligonucleotide probes on the array were designed. The BLASTx searches of NCBI's non-redundant (nr) amino acid sequence and Swiss-Prot databases (E-value <1e-05) were performed using Blast2GO software (BioBam Bioinformatics S.L., Valencia, Spain) (38, 39). Using R and gplots Package, the microarray log_2_ ratios of the identified DEPs were median-centred and subjected to Pearson correlation and complete linkage hierarchical clustering.

The pathway enrichment analyses of different transcript lists (i.e., *R. salmoninarum* infection-responsive transcripts shared between H-BKD and L-BKD groups, infection level-responsive transcripts as well as *R. salmoninarum* infection-responsive transcripts only identified in the H-BKD or L-BKD group) were determined using ClueGO (40) plugin in Cytoscape (v3.5.1) (41). Enrichment (i.e., Right-sided hypergeometric test) analyses were performed using the Gene Ontology database (UniProt: 27.02.2019) for Biological Processes (BPs) and Benjamini-Hochberg test for *p*-value corrections (*p* < 0.05). Furthermore, ClueGO linked the enriched BP GO terms using kappa statistics (42), thus generating networks of functionally-associated terms. Cohen's kappa coefficients are calculated for each term-term relationships based on the shared genes between them. The obtained term-term kappa coefficients were also used to define functional groups of highly-connected terms within the GO networks. The kappa coefficient threshold set for the analysis was 0.4, i.e., term-term relationships with lower coefficients were considered non-significant.

For the subsequent interpretation of the resultant networks, the enriched GO terms were classified, using Gene Ontology Browser (http://www.informatics.jax.org), into 6 functional themes: (1) adaptive immune response; (2) immune response; (3) response to stress; (4) development; (5) metabolic process; and (6) cellular process, localisation, and structure. The GO terms were classified based on the biological process to which they were related and/or their parent terms (especially for highly-specific terms). Briefly, GO terms of biological processes and pathways related to adaptive immunity were classified as “adaptive immune response”. Those immune-related GO terms that could not be classified as “adaptive immune response” were annotated as “immune response”. Other GO terms associated with responses to abiotic and biotic stimuli that are not necessarily involved in immune processes or pathways fell within the “response to stress” theme. GO terms related to tissue development and/or derived from the parent term GO:0032502 (i.e., developmental process) were classified as “development”. GO terms associated with metabolism-related processes and pathways and/or that have the parent term GO:0008152 (i.e., metabolic process) were classified as “metabolic process”. The theme “cellular process, localisation, and structure” grouped all those GO terms not classifiable in any of the previous and derived from either GO:0009987 (i.e., cellular process) or GO:0051179 (i.e., localisation). In a few instances, terms that were close to the root of the ontology could not be assigned to one of the themes (i.e., the term was too general). Finally, some functional groups comprise GO terms from different themes; in such cases, the functional group is coloured according to the theme with the highest number of GO terms.

### qPCR Validation

**Figure 1:**
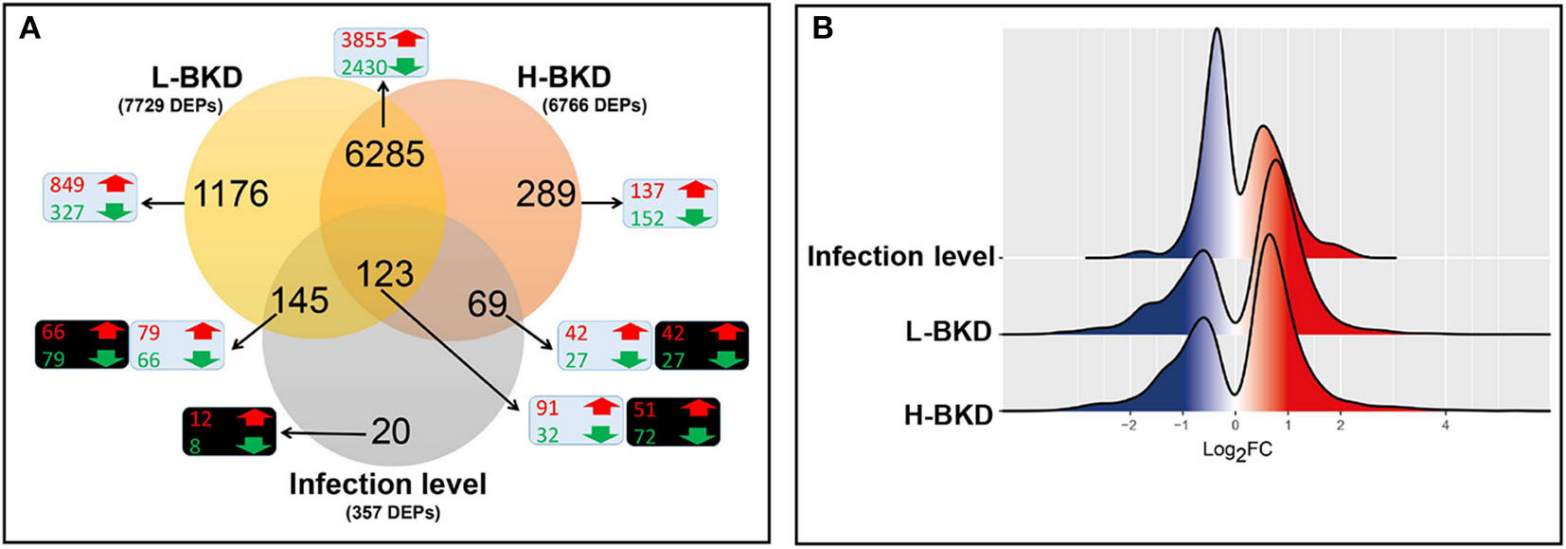
Microarray results of Atlantic salmon head kidney in response to *R. salmoninarum* infection. Fish with no (Control), lower (L-BKD) or higher (H-BKD) level of *R. salmoninarum* infection at 13 dpi were used for microarray analyses (*n* = 5). Fish in the L-BKD and H-BKD groups were infected with the same dose of *R. salmoninarum*, but they showed different levels of infection at 13 dpi, as determined by RNA-based Taq-Man assays. H-BKD: H-BKD vs. Control. L-BKD: L-BKD vs. Control. Infection level: H-BKD vs. L-BKD. **(A)** Overview of microarray results. Differentially expressed probes (DEPs) identified by ANOVA (*p* < 0.01). The number of up-regulated (red) and down-regulated (green) probes in BKD- (L-BKD or H-BKD vs. Control) and infection level (H-BKD vs. L-BKD)-responsive lists are shown in blue and black boxes, respectively. **(B)** Histogram of the frequency density of log_2_-transformed fold-changes (FC) for the DEPs of the different comparisons. Red and blue colours indicate up-regulation and down-regulation, respectively.

A subset of microarray-identified transcripts was subjected to qPCR analysis to test the validity of the microarray results. We selected 35 (i.e., 23 up-regulated and 12 down-regulated) transcripts of interest (TOI) from the BKD-responsive list overlapping only between the L-BKD and H-BKD groups (i.e., 6,285 DEPs; see Figure 1A). Moreover, 10 TOI (i.e., five up-regulated and five down-regulated) were selected from the responsive transcript list specific to the H-BKD group (i.e., 289 DEPs; Figure 1A), whereas 9 TOI (i.e., six up-regulated and three down-regulated) were taken from the L-BKD-specific transcript list (i.e., 1,176 DEPs; Figure 1A). To validate the infection level-responsive gene list, 11 TOI (i.e., six up-regulated and five down-regulated) were selected from transcripts differentially expressed between the H-BKD and L-BKD groups (i.e., total of 357 DEPs; Figure 1A). Among these infection level-responsive transcripts, five TOI (i.e., three up-regulated and two down-regulated) were from the identified transcripts in all comparison (i.e., 123 DEPs), and one down-regulated transcript was selected from the infection level-specific gene list (i.e., 20 DEPs). Also, two (i.e., one up-regulated and one down-regulated) and three (i.e., two up-regulated and one down-regulated) TOI were from the infection level-responsive transcripts overlapping with only-L-BKD and only-H-BKD responsive lists, respectively. Levels of these TOI were measured in samples from all groups (i.e., Control, L-BKD and H-BKD; 15 samples in total). Primers used for qPCR analyses were either designed using Primer3web v4.0.0 (http://primer3.ut.ee/) or taken from previous studies (17, 28, 43–45) (see Supplementary Table S1).

First-strand cDNA templates were synthesised in 20 μl reactions using 1 μg of DNase-treated, column-purified total RNA, nuclease-free water (Invitrogen, Thermo Fisher Scientific), 1 μl of dNTPs (10 mM each; Invitrogen, Thermo Fisher Scientific), random primers (250 ng; Invitrogen, Thermo Fisher Scientific), DTT (10 mM final concentration), first-strand buffer (1X final concentration) and M-MLV reverse transcriptase (200 U; Invitrogen) according to the manufacturer's instructions.

The qPCR assays used in this study were performed following the Minimum Information for Publication of qPCR Experiments (MIQE) guidelines (46). All qPCR assays were conducted in triplicate using the ViiA 7 Real-Time PCR system (384-well format) (Applied Biosystems, Thermo Fisher Scientific), and the qPCR reactions (13 μl) consisted of 6.5 μl Power SYBR Green PCR Master Mix (Applied Biosystems, Thermo Fisher Scientific), 50 nM of each forward and reverse primers (0.52 μl of forward and 0.52 μl of reverse primers), 1.46 μl nuclease-free water (Invitrogen, Thermo Fisher Scientific) and 4 μl cDNA (see below for input total RNA quantity per reaction). The details of the PCR program are described in Eslamloo et al. (47). Two pools were generated using cDNA of all individuals from both BKD groups and control group for primer quality control of up- and down-regulated genes by BKD, respectively. For each primer set (i.e., TOI or normalisers), a 5-point, 3-fold serial dilution of the given cDNA template (i.e., standard curves; starting with cDNA representing 10 ng of input total RNA), as well as a no-template control were used to measure amplification efficiencies. The amplification efficiencies of 10 out of 67 primer pairs were calculated using 4-point serial dilutions of cDNA (see Supplementary Table S1).

Primer pairs used in the current study showed an amplification efficiency (48) ranging between 83 and 110%, an amplicon with a single melting peak and no primer-dimer present in the no-template control (Supplementary Table S1). Firstly, the expression of eight candidate normalisers [i.e., *60S ribosomal protein 32* (*rpl32*), *ef1a1, elongation factor 1 alpha-2* (*ef1a2*), *polyadenylate-binding protein, cytoplasmic 1* (*pabpc1*), *eukaryotic translation initiation factor 3 subunit D* (*eif3d*), *ATP binding cassette sub-family f member 2* (*abcf2*), *RNA polymerase 2* (*polr2*), and *NADH dehydrogenase (ubiquinone) iron-sulfur protein 7* (*ndufs7)*] was measured in all of the experimental samples (five fish per treatment) to determine the most suitable endogenous controls. Thereafter, in the qBase software (49), C_T_ values were analysed by geNorm to calculate the M-value, i.e., a measure of transcript expression stability. Two normaliser transcripts, *ef1a2* and *pabpc1*, showing low M-values (M <0.2) and a comparable expression (i.e., C_T_ values) in all samples were selected for the qPCR assays. Then, the transcript (mRNA) levels of TOI and normalisers were assessed in all 15 samples using cDNA template representing 5 ng of input RNA per PCR reaction as well as a no-template control. The relative quantity (RQ) of each tested transcript was calculated through normalisation to both normaliser transcripts, as implemented by QuantStudio™ Real-Time PCR Software, Relative Quantification Study Application (Version 1.3; Applied Biosystems, Thermo Fisher Scientific). RQ calculations were conducted incorporating the amplification efficiencies of all genes, and RQ value of each transcript was calibrated to the sample that had the lowest normalised gene expression (i.e., assigned an RQ value = 1.0).

All statistical analyses were conducted using the Prism package v7.0 (GraphPad Software Inc., La Jolla, CA). The normality of data (i.e., RQ values) was analysed using the Kolmogorov-Smirnov normality test. Then, One-way ANOVA was applied to identify the differences among groups, followed by Tukey's multiple comparisons *post-hoc* test to determine significant differences (*p* ≤ 0.05) between groups. Kruskal-Wallis test (*p* < 0.05) was used to determine the significant differences between the groups for the transcripts that did not pass the normality test. Furthermore, Pearson's (*r*) correlation was used to test if the expression of transcripts correlated with infection level (RQ values of *R. salmoninarum 16S ribosomal RNA*).

PRIMER 7 (PRIMER-E Ltd., Auckland, New Zealand) was used to identify gene expression patterns among fish groups and TOI via principal coordinates analysis (PCoA) based on a Bray-Curtis similarity matrix generated using RQ values of TOI as well as infection level values (i.e., RQ values of *16S ribosomal RNA* of *R. salmoninarum* normalised to Atlantic salmon *ef1a1* and calibrated to the sample with the lowest normalised expression level).

## Results

### Microarray Analyses

**Figure 2:**
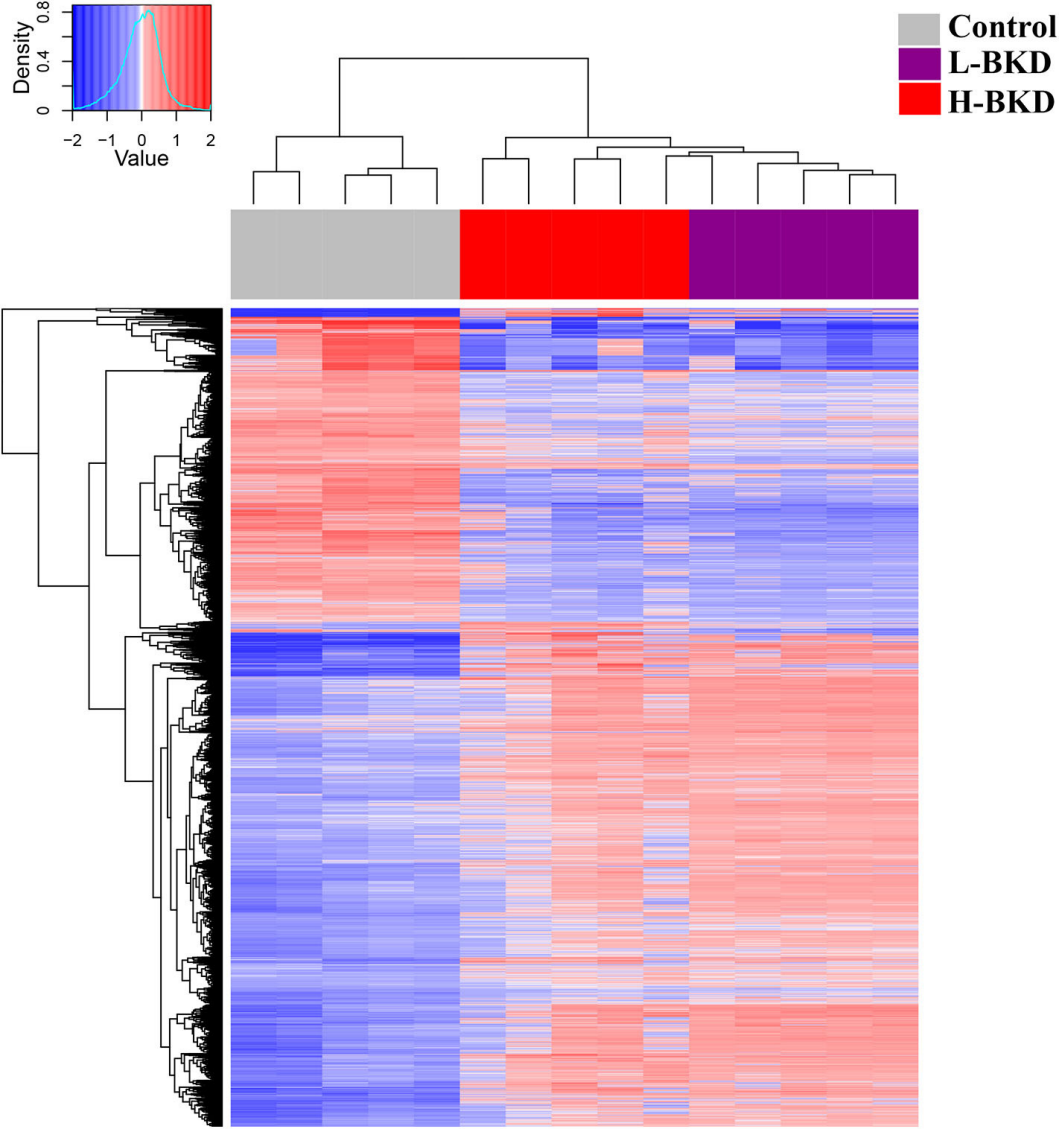
Hierarchical clustering analyses of microarray results. Fish with no (Control), lower (L-BKD), or higher (H-BKD) level of *R. salmoninarum* infection at 13 dpi were used for microarray analyses (*n* = 5). All the differentially expressed probes (DEPs) identified by ANOVA (*p* < 0.01) were used for clustering analyses.

In this study, we used a 44K microarray platform to profile the response of Atlantic salmon head kidney to BKD. We compared the transcriptome profile of fish showing a lower (L-BKD) or a higher (H-BKD) level of the *R. salmoninarum* infection together and with a control group. The L-BKD and H-BKD fish were injected with the same dose of live *R. salmoninarum*, but they showed various infection levels, which also indicate the fish susceptibility to *R. salmoninarum*, at 13 dpi, as determined by TaqMan assays. Figure 1A illustrates the overall results of the microarray analyses in the present study. Using one-way ANOVA (*p* ≤ 0.01), 6,766 DEPs were identified in the H-BKD group, whereas 7,729 DEPs were found in the L-BKD group compared to the control. When the H-BKD and L-BKD groups were compared, there were 357 DEPs significantly affected by the infection level (i.e., H-BKD vs. L-BKD; 171 up-regulated and 186 down-regulated). As shown by the Venn diagrams (Figure 1A), all three comparisons in the present study shared 123 DEPs. There were 6,408 DEPs (6,285 + 123 DEPs: 3,946 up-regulated and 2,462 down-regulated) overlapping between the H-BKD and L-BKD transcript lists. Among the infection level-responsive transcripts, 69 and 145 DEPs overlapped with BKD-responsive probes identified only in the H-BKD and L-BKD groups, respectively. Supplementary Table S2 shows the complete list of the identified DEPs. As shown by Figure 1A, there was a larger number of BKD-responsive probes in the L-BKD group compared with the H-BKD group. Furthermore, the up-regulated probes in the L-BKD group were distributed slightly more frequently over higher log_2_ fold-changes compared with those in the H-BKD group (Figure 1B). Using all identified DEPs (ANOVA; *p* ≤ 0.01) in hierarchical clustering analyses, samples associated with a given group were closely clustered (Figure 2), reflecting that samples in each group share an overall comparable gene expression response.

### Pathway Enrichment Analyses

**Figure 3:**
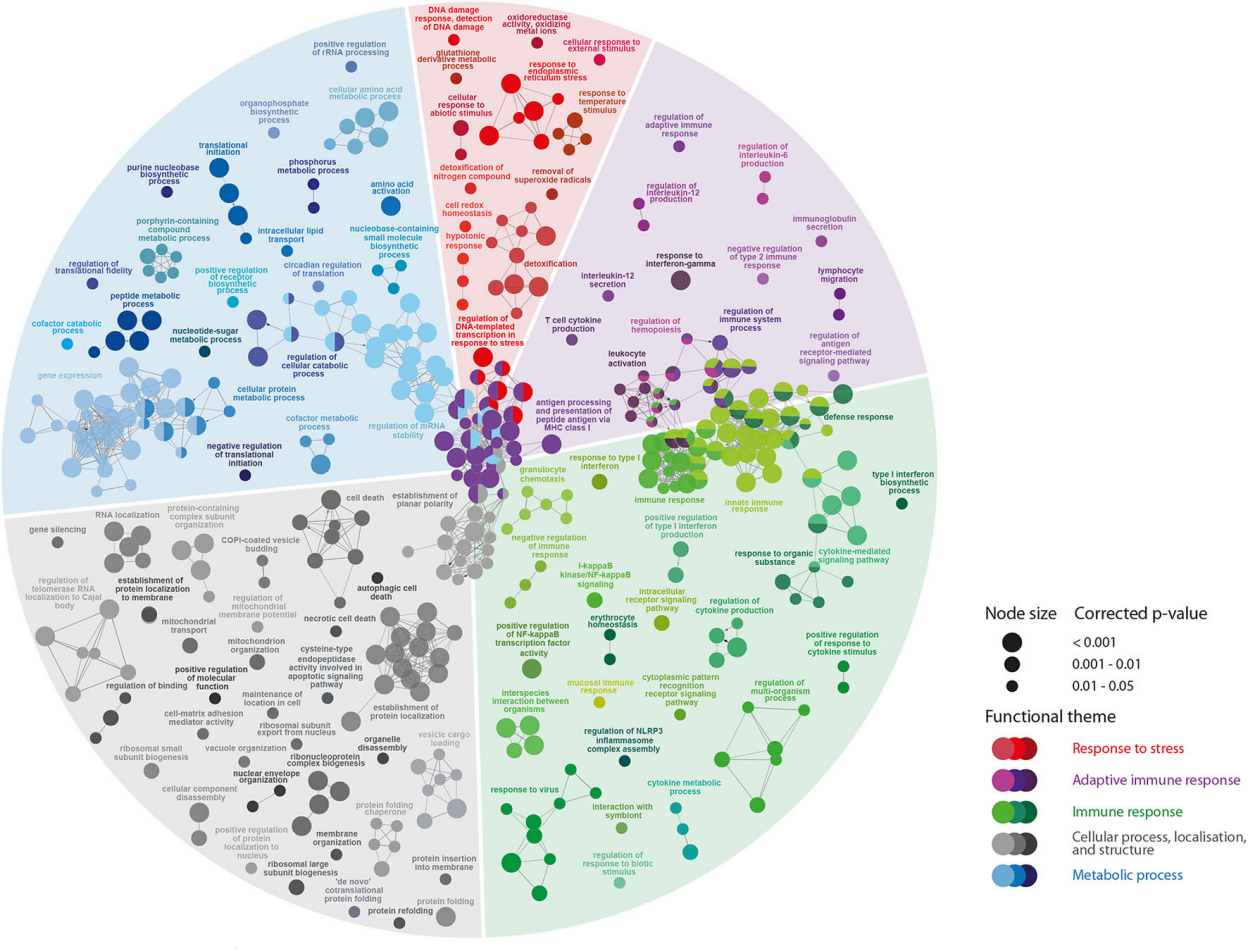
The pathway enrichment analyses of *R. salmoninarum* infection-responsive transcripts shared by both BKD groups. Nodes represent significantly enriched GO terms [right-sided hypergeometric test, *p*-values (*p* < 0.05) corrected by Benjamini-Hochberg]. Nodes are coloured according to the functional theme to which they were assigned. Node size corresponds to different *p*-value ranges (i.e., *p* < 0.001, 0.001–0.01, and 0.01–0.05). Highly related terms (kappa coefficient > 0.4) are connected with grey lines. Individual GO terms (i.e., single node) and GO networks (i.e., multiple connected nodes) are grouped by functional theme and arranged to fit the sectors of a pie chart representing the proportion of GO terms in each functional theme.

We used ClueGO to identify the BPs over-represented in BKD-responsive transcript lists compared to the whole microarray platform. First, we tested the BPs enriched in BKD-responsive transcripts overlapping between both treatments (6,408 DEPs). The enriched BPs by BKD (Figure 3) were associated with response to stress (8.6%), adaptive immunity (15.5%), immune responses (27.6%), cellular processes (23.8%) and metabolic process (24.3%).

Several pathways related to adaptive immunity were dysregulated by *R. salmoninarum* infection in Atlantic salmon (Figure 3; Supplementary Table S3A). This includes the induction of pathways linked to lymphocyte differentiation or activation (e.g., T-cell activation, regulation of lymphocyte activation and lymphocyte migration, immunoglobulin secretion), adaptive immunity-related cytokine responses (e.g., NIK/NF-kappaB signalling, interleukin-12 secretion, response to interferon-gamma, regulation of interleukin-6 production, T-cell cytokine production) and antigen presentation processes (e.g., antigen processing and presentation of peptide antigen via MHC class I). A large number of BPs involved in immune response (e.g., defence response, activation of the innate immune response, inflammatory response, response to molecule of bacterial origin, defence response to virus) and regulation of immune responses (e.g., regulation of NLRP3 inflammasome complex assembly, negative regulation of the viral process, immune response-regulating signalling pathway, regulation of innate immune response) were activated in response to BKD. Furthermore, we identified BKD-triggered dysregulation of several molecular (e.g., type I interferon production, cytokine production, response to cytokine, immune effector process) and cellular (e.g., leukocyte activation, granulocyte activation, leukocyte chemotaxis, regulation of leukocyte migration) responses related to innate immunity (Figure 3; Supplementary Table S3A). There was extensive dysregulation of pathways related to cellular processes (e.g., necrotic cell death, negative regulation of cell death, programmed cell death, autophagic cell death) in *R. salmoninarum*-infected Atlantic salmon. Further, our findings show that BKD caused a massive metabolic dysregulation, such as nucleotide- (e.g., regulation of gene expression, regulation of mRNA stability, regulation of nucleotide metabolic process) and protein-related (e.g., cellular amino acid metabolic process, protein metabolic process, amino acid activation, translational initiation) processes, in the Atlantic salmon head kidney (Figure 3).

**Figure 4:**
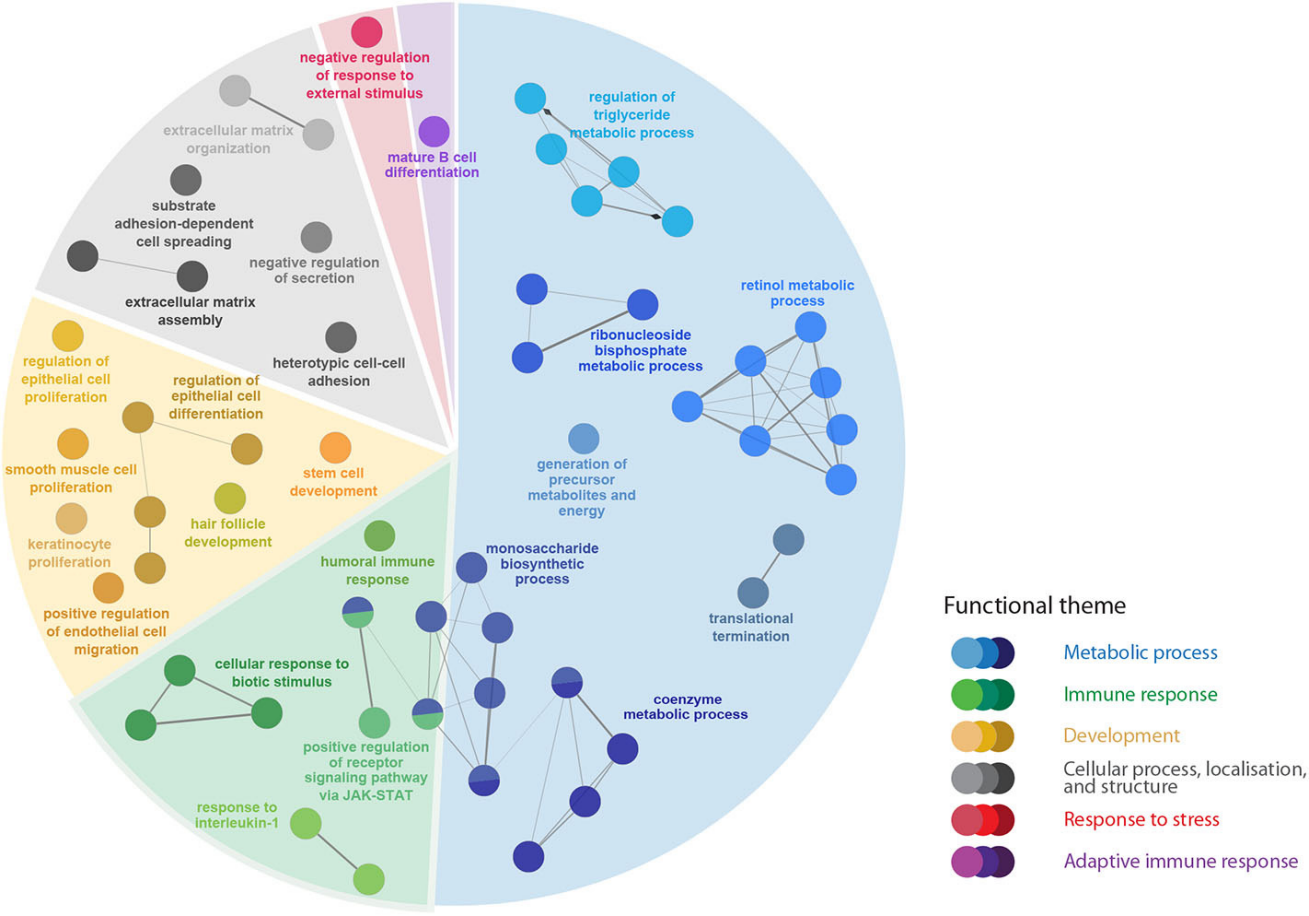
The pathway enrichment analyses of transcripts responsive to the *R. salmoninarum* infection level (H-BKD vs. L-BKD). Nodes represent significantly enriched GO terms [right-sided hypergeometric test, *p*-values (*p* < 0.05) corrected by Benjamini-Hochberg]. Nodes are coloured according to the functional theme to which they were assigned. Highly related terms (kappa coefficient > 0.4) are connected with grey lines. Individual GO terms (i.e., single node) and GO networks (i.e., multiple connected nodes) are grouped by functional theme and arranged to fit the sectors of a pie chart representing the proportion of GO terms in each functional theme.

There were 357 DEPs by infection level (i.e., H-BKD vs. L-BKD), which were associated with several BPs including the regulation of Janus kinase (JAK)-Signal transducer and activator of transcription (STAT) signalling, activated downstream of the interferon (IFN) pathway (Figure 4; Supplementary Table S3B). Further, infection level-responsive transcripts were involved in humoral immune responses, B-cell differentiation, as well as response to interleukin-1 (Figure 4). Our findings showed that the infection level influenced multiple metabolic pathways in *R. salmoninarum*-infected Atlantic salmon.

There were 1,321 DEPs only identified in the L-BKD group (L-BKD vs. Control), and their over-represented BPs were associated with gene expression and its regulation, as well as metabolic processes. In addition, these DEPs were associated with viral process and activation of immune-related myeloid cells (Supplementary Table S3C). Several immune-related BPs were over-represented in the 358 DEPs only identified in the H-BKD group (H-BKD vs. Control; Supplementary Table S3D). Pathways involved in the regulation of immune processes, defence response, cell surface receptor signalling, cell communication and regulation of cell differentiation were enriched in the transcript list only identified in the H-BKD group (Supplementary Table S3D).

### qPCR Validation

Sixty-five transcripts representing various molecular pathways (e.g., innate and adaptive immune responses) and dysregulations (i.e., up- and down-regulation, high and low fold-changes) were selected for qPCR validation. To have an acceptable representation of the microarray results, the transcripts contributing to qPCR assays were from different comparisons and gene lists, including the transcript lists overlapping between groups or specific to a given group. All the studied transcripts, except for *fc receptor-like protein 5* (*fcrl5*), showed the same fold-change direction as the microarray results of the differentially expressed transcripts (Supplementary Table S4); however, for some transcripts, the differences were not significant by qPCR. There were 35 (12 down-regulated and 23 up-regulated transcripts in response to BKD) qPCR-studied transcripts selected from the 6,285 DEPs overlapping only between L-BKD and H-BKD group transcript lists (Figure 1A), and except for *major histocompatibility class I* (*mh1*), the microarray results were confirmed (*p* < 0.05) by qPCR for all of the transcripts for at least one of the groups. Nine (4 down-regulated and 5 up-regulated) transcripts were from the L-BKD-specific transcript list (i.e., 1,176 DEPs; Figure 1A), and the microarray results of these transcripts were confirmed (*p* < 0.05) for all of them except for *fcrl5* and *dual specificity protein phosphatase 7* (*dusp7*). qPCR assays confirmed (*p* < 0.05) the results of 10 transcripts (5 down-regulated and 5 up-regulated) selected from the H-BKD-specific transcript list (i.e., 289 DEPs; Figure 1A). Despite showing the same direction of fold-changes (Supplementary Table S4), microarray results were only confirmed to be significantly different for 6 [i.e., *guanine deaminase* (*gda*), *granzyme a precursor* (*gzma*), *interferon-induced very large GTPase 1* (*gvinp1*), *leukemia inhibitory factor receptor* (*lifr*)*, leucine-rich repeat transmembrane protein FLRT3* (*flrt3*), *interleukin 13 receptor alpha 1b* (*il13ra1b*)] out of 11 (5 down-regulated and 6 up-regulated) qPCR-studied transcripts selected from the infection level-responsive transcript list (i.e., H-BKD vs. L-BKD: 357 DEPs; Figure 1A). We categorised the qPCR-studied transcripts based on their putative function in immune responses.

**Figure 5:**
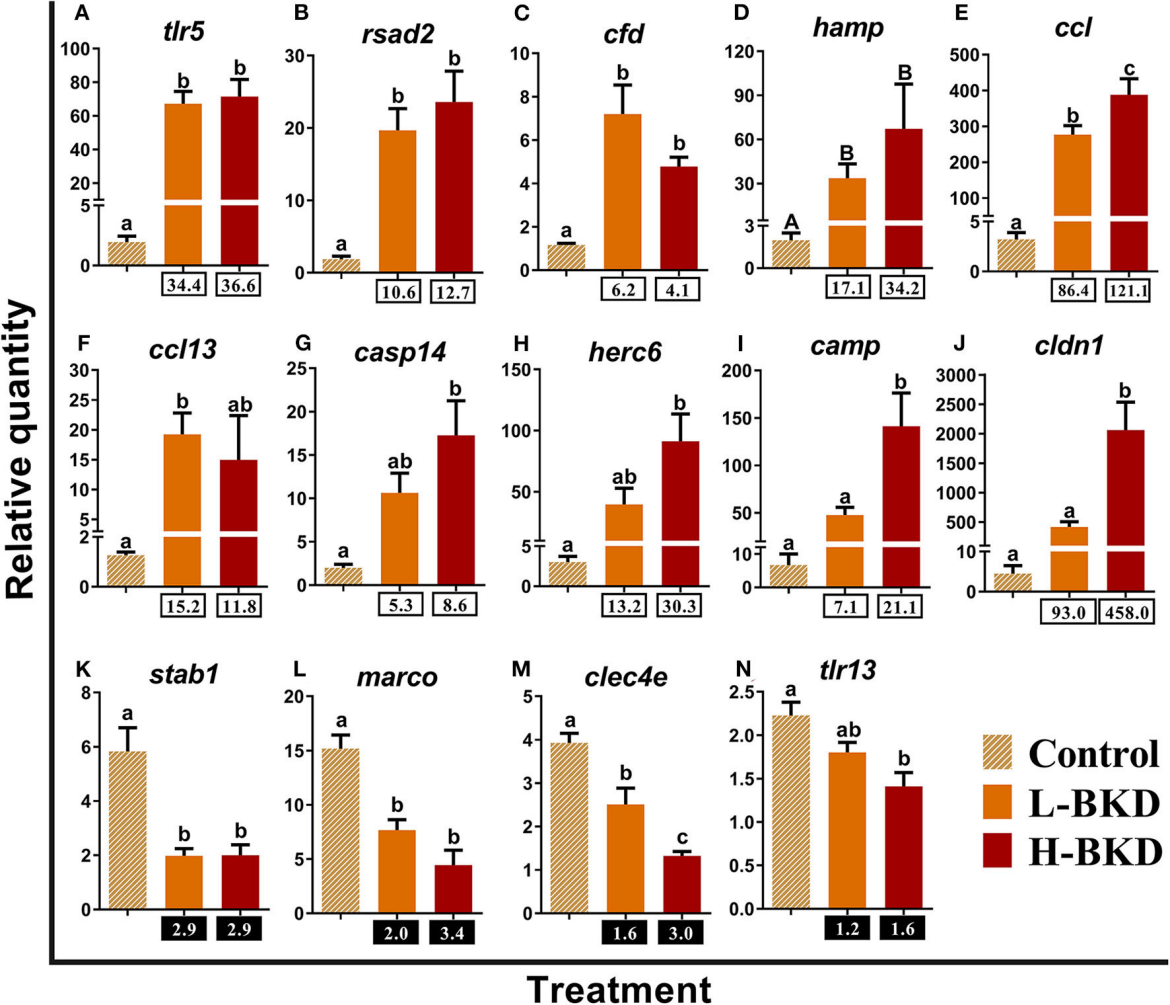
**(A–N)** qPCR for *R. salmoninarum* infection-responsive transcripts playing putative roles in innate immune response. Fish with no (Control), lower (L-BKD) or higher (H-BKD) level of *R. salmoninarum* infection at 13 dpi were used for qPCR validation (*n* = 5). Fish in the L-BKD and H-BKD groups were infected with the same dose of *R. salmoninarum*, but they showed different levels of infection at 13 dpi, as determined by RNA-based Taq-Man assays. Data are presented as mean ± SE, with the lowest expressing sample as calibrator [i.e., set to relative quantity (RQ) 1.0]. Lower-case letters indicate significant differences between groups, as determined by one-way ANOVA with Tukey's *post-hoc* test (*p* < 0.05). Upper-case letters indicate significant differences between groups, as determined by Kruskal-Wallis test (*p* < 0.05). Fold-changes are shown below H-BKD and L-BKD groups, calculated as (mean H-BKD or L-BKD RQ)/(mean control RQ). Black boxes indicate the down-regulated or negative fold-changes, which were calculated as 1/fold-change for comparisons that yielded fold-change values <1.

qPCR results of 14 transcripts playing roles in innate immune responses are shown in Figure 5. There was significant up-regulation of *toll-like receptor 5* (*tlr5*)*, radical s-adenosyl methionine domain containing* (*rsad2*; alias *viperin*), *complement factor D precursor* (*cfd*), and *hepcidin antimicrobial peptide* (*hamp*) in both L-BKD and H-BKD groups compared to the control (Figures 5A–D). Transcript expression of *CC chemokine* (*ccl*) increased in both L-BKD and H-BKD groups, but its level was significantly higher (*p* < 0.05) in the H-BKD group compared to the L-BKD group (Figure 5E). Despite up-regulation in both treatments, a significant difference for *C-C motif chemokine 13* (*ccl13*) expression was only seen between L-BKD compared with the control (Figure 5F). On the other hand, the significant up-regulation compared to the control for *caspase-14* (*casp14*), *E3 ubiquitin-protein ligase herc6* (*herc6*)*, cathelicidin antimicrobial peptide* (*camp*), and *claudin-1* (*cldn1*) was only seen for the H-BKD, and *camp* and *cldn1* levels in the H-BKD group were significantly higher than those in the L-BKD group (Figures 5G–J). *R. salmoninarum* infection down-regulated the levels of *stabilin-1* (*stab1*)*, macrophage receptor with collagenous structure* (*marco*), and *c-type lectin domain family 4 member e* (*clec4e*) in both H-BKD and L-BKD groups (Figures 5K–M). There was an infection level-dependent down-regulation for *clec4e*, with the lowest expression in the H-BKD group. Also, *toll-like receptor 13* (*tlr13*) was only down-regulated in the H-BKD group, compared to the control (Figure 5N).

**Figure 6:**
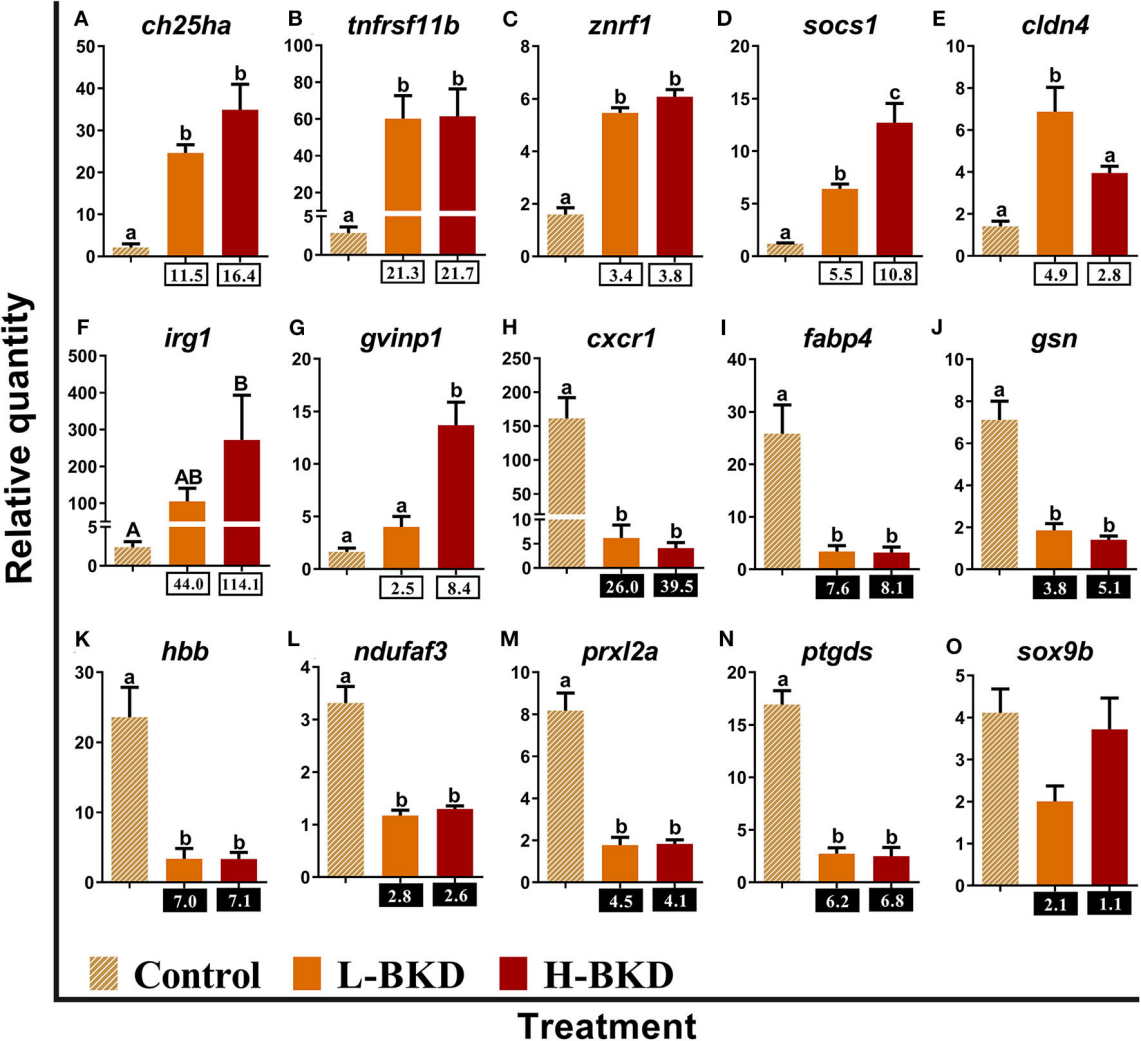
**(A–O)** qPCR for *R. salmoninarum* infection-responsive transcripts involved in the regulation of innate immune and inflammatory responses. Fish with no (Control), lower (L-BKD) or higher (H-BKD) level of *R. salmoninarum* infection at 13 dpi were used for qPCR validation (*n* = 5). Data are presented as mean ± SE, with the lowest expressing sample as calibrator [i.e., set to relative quantity (RQ) 1.0]. Lower-case letters indicate significant differences between groups, as determined by one-way ANOVA with Tukey's *post-hoc* test (*p* < 0.05). Upper-case letters indicate significant differences between groups, as determined by Kruskal-Wallis test (*p* < 0.05). Fold-changes are shown below H-BKD and L-BKD groups, calculated as (mean H-BKD or L-BKD RQ)/(mean control RQ). Black boxes indicate the down-regulated or negative fold-changes, which were calculated as 1/fold-change for comparisons that yielded fold-change values <1.

Fifteen identified BKD-responsive transcripts involved in the regulation of innate immune and inflammatory responses were subjected to qPCR validation (Figure 6). The levels of *cholesterol 25-hydroxylase-like protein a* (*ch25ha*)*, tumor necrosis factor receptor superfamily member 11b* (*tnfrsf11b*)*, E3 ubiquitin-protein ligase znrf1* (*znrf1*), and *suppressor of cytokine signalling 1* (*socs1*) were significantly induced by *R. salmoninarum* infection in both H-BKD and L-BKD groups, and there was an infection level-dependent induction for *socs1*, with the highest level seen for the H-BKD group (Figures 6A–D). There was an up-regulation of *claudin 4* (*cldn4*) in the L-BKD group compared to both H-BKD and control groups (Figure 6E). On the contrary, BKD-induced expression of *immune-responsive gene 1* (*irg1*) and *gvinp1* was only found in the H-BKD group, and *gvinp1* level was significantly higher in the H-BKD group, compared to the L-BKD group (Figures 6F,G). *R. salmoninarum* infection repressed the expression of *C-X-C chemokine receptor type 1* (*cxcr1*), *fatty acid-binding protein 4, adipocyte* (*fabp4*)*, gelsolin* (*gsn*)*, haemoglobin subunit beta* (*hbb*)*, NADH dehydrogenase [ubiquinone] 1 alpha subcomplex assembly factor 3* (*ndufaf3*)*, peroxiredoxin-like 2a* (*prxl2a*), and *prostaglandin D2 synthase* (*ptgds*) in both H-BKD and L-BKD groups (Figures 6H–N). Microarray results were not confirmed (*p* > 0.05) for *transcription factor Sox-9-b* (*sox9b*); its expression did not change among treatments (Figure 6O).

**Figure 7:**
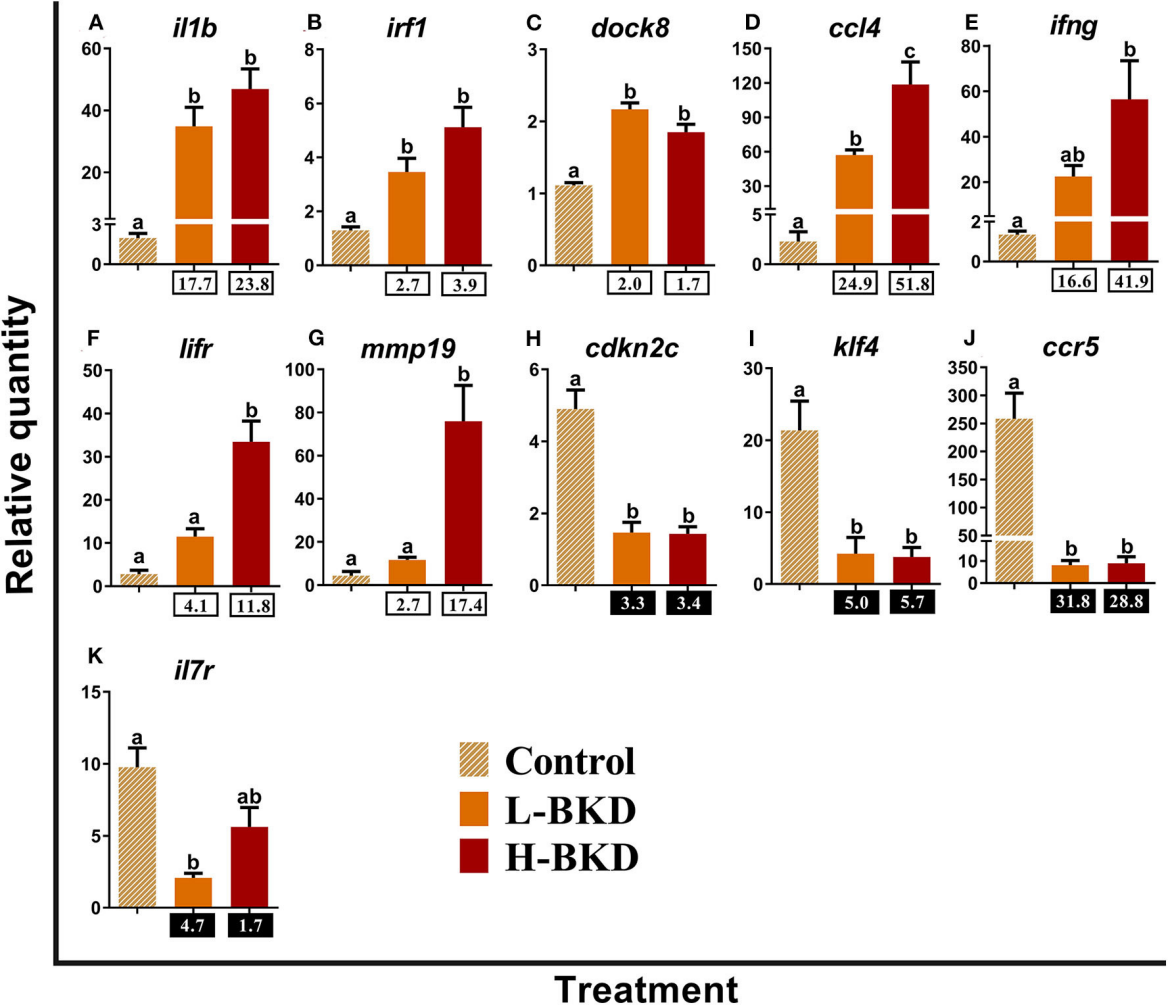
**(A–K)** qPCR for *R. salmoninarum* infection-responsive transcripts playing molecular roles as receptors or immune effectors in lymphocyte differentiation. Fish with no (Control), lower (L-BKD) or higher (H-BKD) level of *R. salmoninarum* infection at 13 dpi were used for qPCR validation (*n* = 5). Data are presented as mean ± SE, with the lowest expressing sample as calibrator [i.e., set to relative quantity (RQ) 1.0]. Lower-case letters indicate significant differences between groups, as determined by one-way ANOVA with Tukey's *post-hoc* test (*p* < 0.05). Fold-changes are shown below H-BKD and L-BKD groups, calculated as (mean H-BKD or L-BKD RQ)/(mean control RQ). Black boxes indicate the down-regulated or negative fold-changes, which were calculated as 1/fold-change for comparisons that yielded fold-change values <1.

Eleven transcripts studied by qPCR in the current study play molecular roles as receptors or immune effectors in lymphocyte differentiation (Figure 7). Induction of *interleukin-1 beta* (*il1b*), *interferon regulatory factor 1* (*irf1*), *dedicator of cytokinesis protein 8* (*dock8*), and *C-C motif chemokine 4* (*ccl4*) occurred in both H-BKD and L-BKD groups, but *ccl4* level was significantly higher in the H-BKD compared to the L-BKD group (Figures 7A–D). However, significant up-regulation of *interferon gamma* (*ifng*) in response to *R. salmoninarum* infection was only seen in the H-BKD group (Figure 7E). Moreover, *lifr* and *matrix metallopeptidase-19* (*mmp19*) expression in the H-BKD group was significantly higher (*p* < 0.05) than that in the other groups, and there was not a significant difference between the L-BKD and control groups in expression of these transcripts (Figures 7F,G). *R. salmoninarum* infection suppressed the expression of *cyclin-dependent kinase inhibitor 2c* (*cdkn2c*)*, kruppel-like factor 4* (*klf4*), and *C-C chemokine receptor type 5* (*ccr5*) in both H-BKD and L-BKD groups (Figures 7H–J). As in the microarray results, BKD-dependent down-regulation of *interleukin-7 receptor subunit alpha* (*il7r*) was only observed in the L-BKD group; the difference between control and H-BKD groups for this transcript was not significant (Figure 7K).

**Figure 8:**
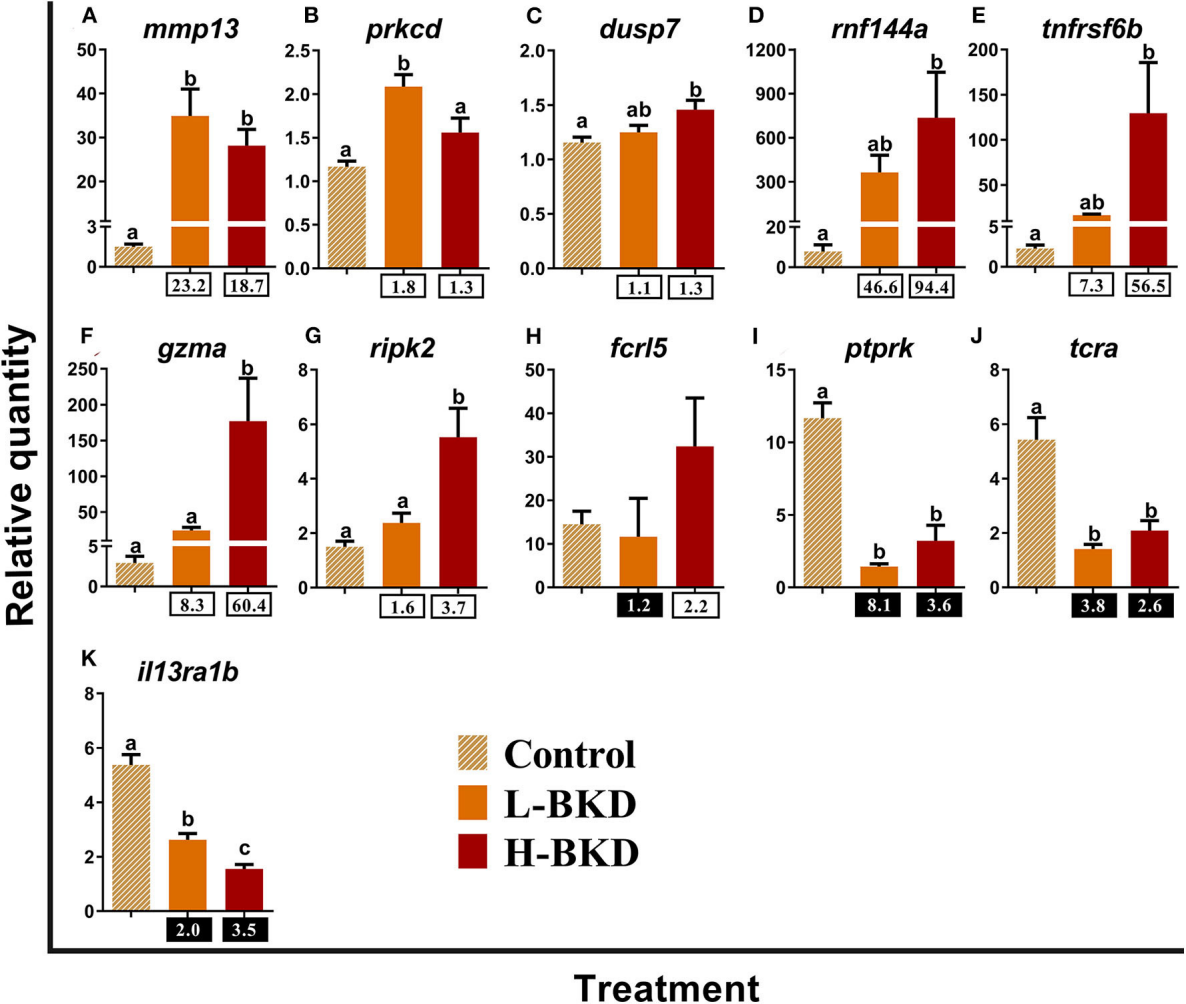
**(A–K)** qPCR for *R. salmoninarum* infection-responsive transcripts playing putative roles in lymphocyte functions. Fish with no (Control), lower (L-BKD), or higher (H-BKD) level of *R. salmoninarum* infection at 13 dpi were used for qPCR validation (*n* = 5). Data are presented as mean ± SE, with the lowest expressing sample as calibrator [i.e., set to relative quantity (RQ) 1.0]. Lower-case letters indicate significant differences between groups, as determined by one-way ANOVA with Tukey's *post-hoc* test (*p* < 0.05). Fold-changes are shown below H-BKD and L-BKD groups, calculated as (mean H-BKD or L-BKD RQ)/(mean control RQ). Black boxes indicate the down-regulated or negative fold-changes, which were calculated as 1/fold-change for comparisons that yielded fold-change values <1.

We studied the expression levels of 11 transcripts encoding proteins that play putative roles in lymphocyte functions (Figure 8). Transcript levels of *matrix metallopeptidase-13* (*mmp13*) were up-regulated in response to *R. salmoninarum* infection in both L-BKD and H-BKD groups (Figure 8A). The expression levels of *protein kinase c delta type* (*prkcd*) and *dusp7* slightly but significantly increased (*p* < 0.05) only in the L-BKD and H-BKD groups, respectively (Figures 8B,C). Moreover, *E3 ubiquitin-protein ligase RNF144a-a* (*rnf144a*) and *tumor necrosis factor receptor superfamily member 6b* (*tnfrsf6b*) were strongly induced by *R. salmoninarum* infection only in the H-BKD group; the changes between the L-BKD and control groups were not significant (Figures 8D,E). Similar results were seen for *gzma* and *receptor-interacting serine/threonine-protein kinase 2* (*ripk2*), even though these transcripts showed significantly higher levels in the H-BKD compared with the L-BKD group (Figures 8F,G). The microarray results were not validated for *fcrl5*, as there was no difference among treatments for this transcript (Figure 8H). *R. salmoninarum* infection suppressed the levels of *receptor-type tyrosine-protein phosphatase kappa-like* (*ptprk*)*, t-cell receptor alpha* (*tcra*) and *il13ra1b* in both L-BKD and H-BKD groups, although there was an infection level-dependent suppression for *il13ra1b*, with the maximum down-regulation in the H-BKD group (Figures 8I–K).

**Figure 9:**
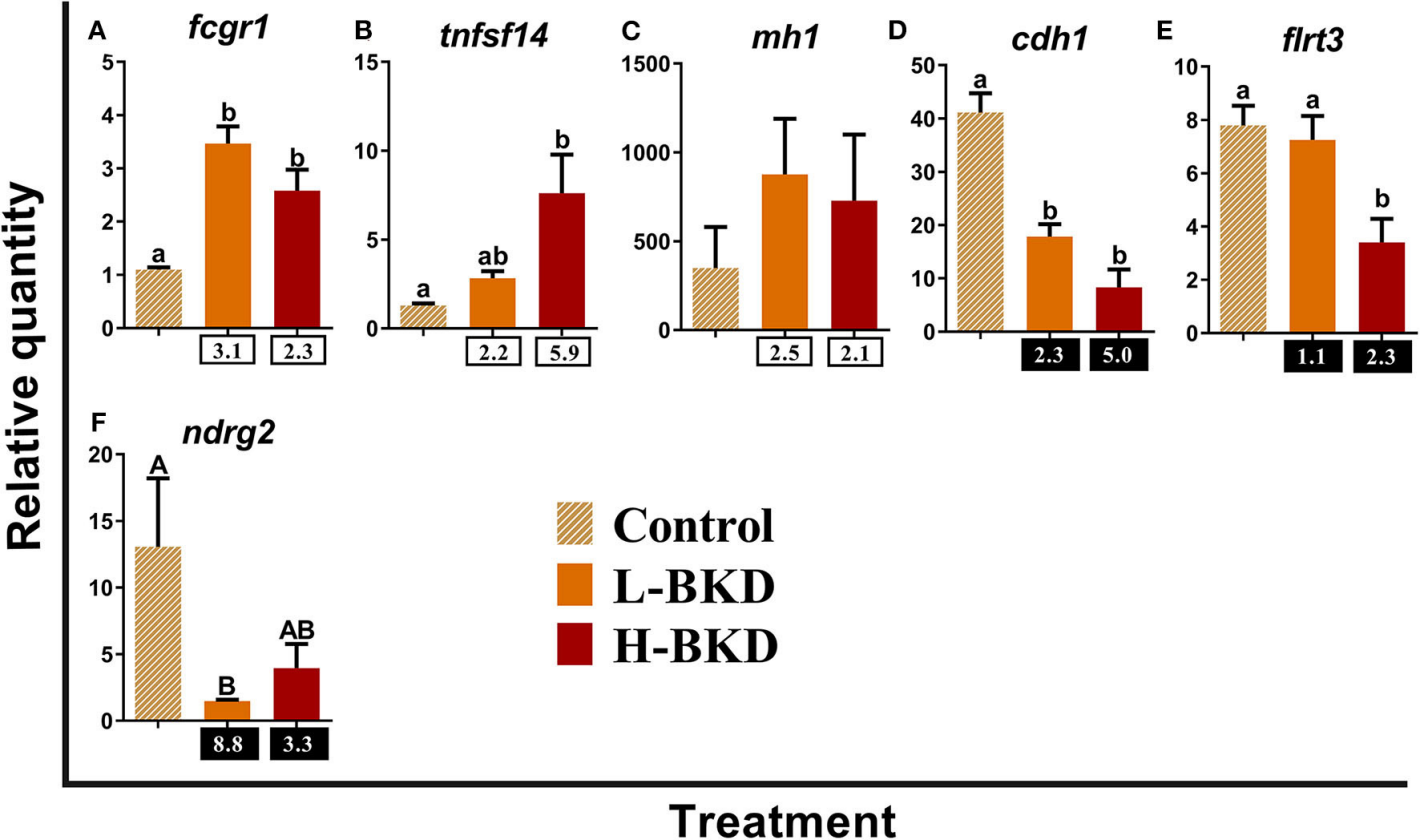
**(A–F)** qPCR for *R. salmoninarum* infection-responsive transcripts involved in antigen-presenting cell functions. Fish with no (Control), lower (L-BKD) or higher (H-BKD) level of *R. salmoninarum* infection at 13 dpi were used for qPCR validation (*n* = 5). Data are presented as mean ± SE, with the lowest expressing sample as calibrator [i.e., set to relative quantity (RQ) 1.0]. Lower-case letters indicate significant differences between groups, as determined by one-way ANOVA with Tukey's *post-hoc* test (*p* < 0.05). Upper-case letters indicate significant differences between groups, as determined by Kruskal-Wallis test (*p* < 0.05). Fold-changes are shown below H-BKD and L-BKD groups, calculated as (mean H-BKD or L-BKD RQ)/(mean control RQ). Black boxes indicate the down-regulated or negative fold-changes, which were calculated as 1/fold-change for comparisons that yielded fold-change values <1.

Figure 9 shows the qPCR results of the 6 transcripts involved in antigen-presenting cell (APC) functions. While *high affinity immunoglobulin gamma Fc receptor I* (*fcgr1*) was up-regulated in both L-BKD and H-BKD groups in response to *R. salmoninarum* infection, *tumor necrosis factor ligand superfamily member 14* (*tnfsf14*) induction was only seen in the H-BKD group (Figures 9A,B). Significant difference among treatments was not found for *mh1* (Figure 9C). *R. salmoninarum* infection down-regulated the expression of *B-cadherin-like* (*cdh1*) in both BKD conditions (Figure 9D). However, *R. salmoninarum* infection-suppressed expression of *flrt3* and *n-myc downstream-regulated gene* (*ndrg2*) was only seen in the H-BKD and L-BKD group, respectively (Figures 9E,F).

**Figure 10:**
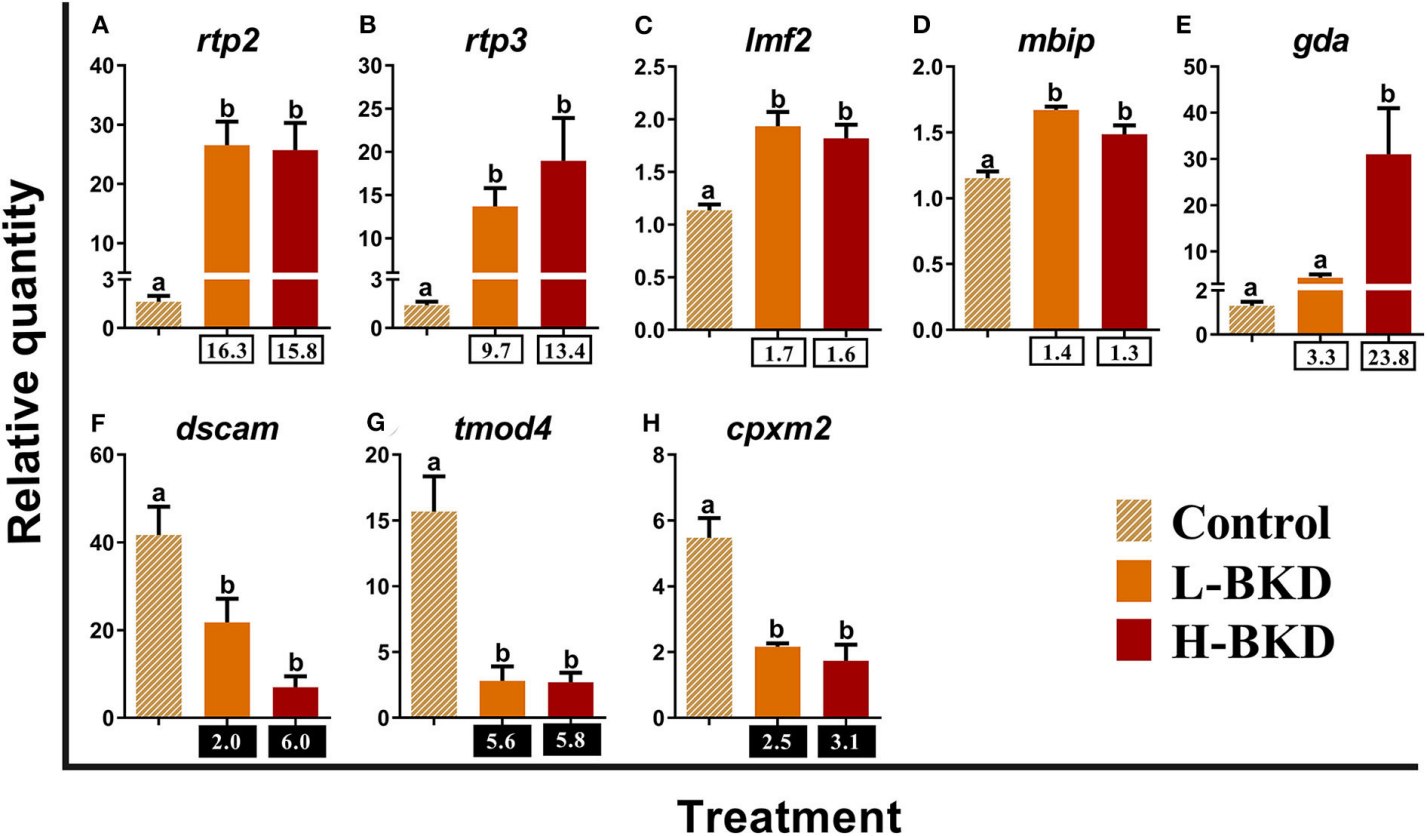
**(A–H)** qPCR for *R. salmoninarum* infection-responsive transcripts with unknown function in immune responses of mammalian and fish species. Fish with no (Control), lower (L-BKD), or higher (H-BKD) level of *R. salmoninarum* infection at 13 dpi were used for qPCR validation (*n* = 5). Data are presented as mean ± SE, with the lowest expressing sample as calibrator [i.e., set to relative quantity (RQ) 1.0]. Lower-case letters indicate significant differences between groups, as determined by one-way ANOVA with Tukey's *post-hoc* test (*p* < 0.05). Fold-changes are shown below H-BKD and L-BKD groups, calculated as (mean H-BKD or L-BKD RQ)/(mean control RQ). Black boxes indicate the down-regulated or negative fold-changes, which were calculated as 1/fold-change for comparisons that yielded fold-change values <1.

In addition to immune response-associated transcripts, we studied 8 transcripts with unknown function in immune responses of mammalian and fish species (Figure 10). The levels of *receptor-transporting protein 2* (*rtp2*)*, receptor-transporting protein 3* (*rtp3*)*, lipase maturation factor 2* (*lmf2*), and *MAP3K12-binding inhibitory protein 1* (*mbip*) were up-regulated by *R. salmoninarum* infection in both L-BKD and H-BKD treatments (Figures 10A–D). Up-regulation of *gda* was observed in the H-BKD group compared to both L-BKD and control groups (Figure 10E). On the other hand, *down syndrome cell adhesion molecule* (*dscam*)*, tropomodulin-4-like* (*tmod4*), and *inactive carboxypeptidase-like protein X2* (*cpxm2*) expression was suppressed in response to *R. salmoninarum* infection in both L-BKD and H-BKD groups (Figures 10F–H).

Among all the qPCR-studied transcripts, 27 transcripts showed a significant correlation with the level of *R. salmoninarum* infection (Supplementary Table S5). The RQ values of *lifr, herc6, gzma, mmp19, gda, gvinp1, rtp3, socs1, casp14, dusp7, cldn1, camp, rsad2, ccl, irf1, ifng, ccl4, ripk2, rtp2, tnfsf14*, and *fcrl5* were positively correlated with the infection level (RQ value of *R. salmoninarum* expression), whereas *il13ra1b, dscam, cdh1, clec4e, marco*, and *flrt3* showed a significant negative correlation with the infection level. The significant correlations of these transcripts with the infection level suggest them as suitable biomarkers for assessing the *R. salmoninarum* level-dependent responses in Atlantic salmon.

**Figure 11:**
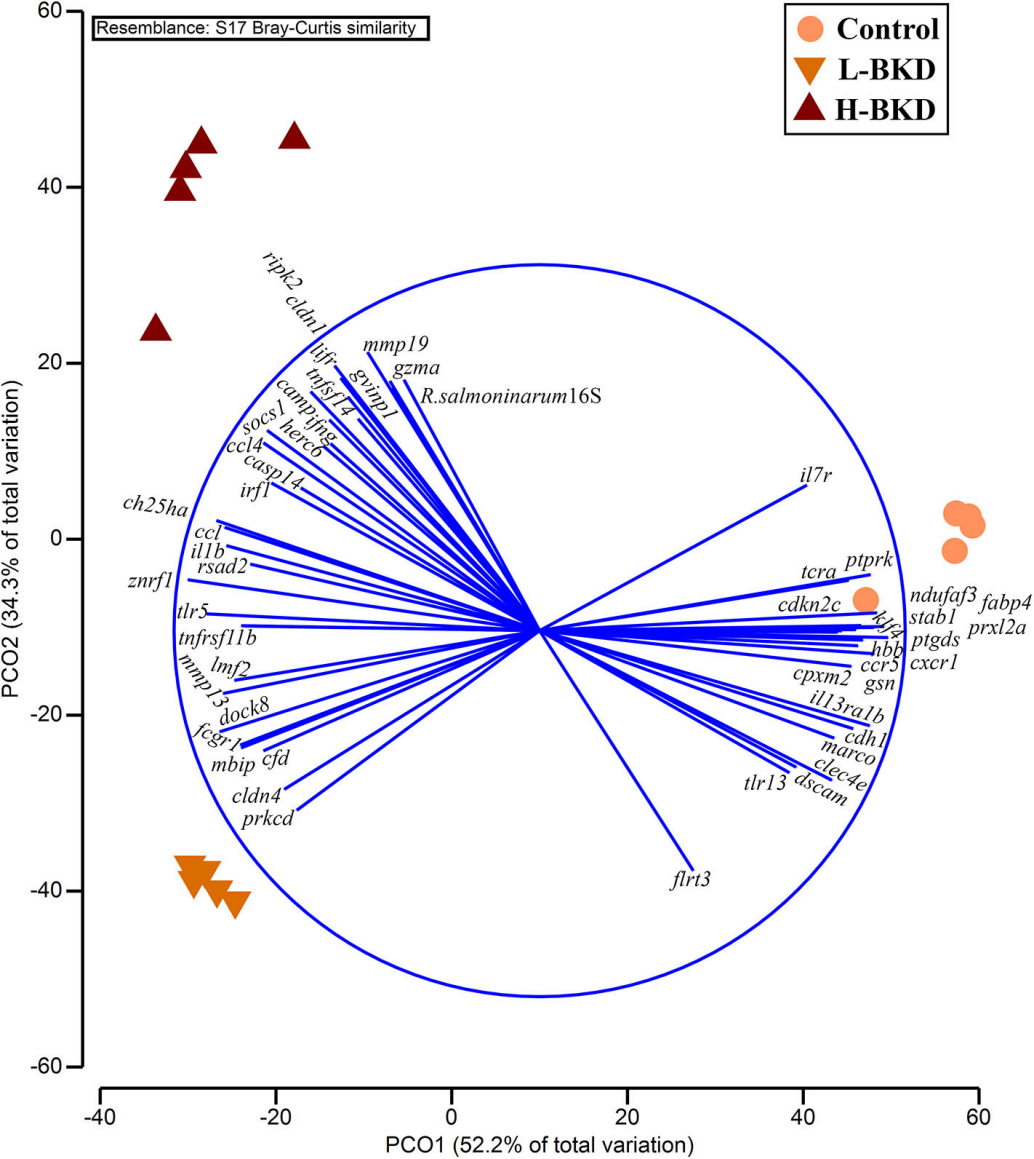
Principal coordinate analysis using all qPCR-studied transcripts and *R. salmoninarum* infection level. Fish with no (Control), lower (L-BKD) or higher (H-BKD) level of *R. salmoninarum* infection at 13 dpi were used for qPCR validation (*n* = 5). Fish in the L-BKD and H-BKD groups were infected with the same dose of *R. salmoninarum*, but they showed different levels of infection at 13 dpi. The infection level indicates the expression of *R. salmoninarum 16S ribosomal RNA* as determined by RNA-based TaqMan assays. All vectors with Pearson's *r* < 0.75 are shown.

The PCoA showed a clear separation among the experimental groups (Figure 11). PCO1 (i.e., 52.2% of total variation) and PCO2 (i.e., 34.3% of total variation) collectively explain 86.5% of the total variation. The control individuals were positively plotted on the PCO1, whereas individuals associated with both L-BKD and H-BKD groups negatively loaded on the PCO1. Samples were separated based on the infection level on PCO2, as the H-BKD and L-BKD individuals were loaded positively and negatively, respectively, on PCO2. Although all infected samples were associated with the left side vectors (e.g., *mmp19, gvinp1, cldn1, lifr, socs1, ccl4, ccl, ch25ha, il1b, znrf1, tlr5, mmp13, dock8, fcgr1*, and *rsad2*), varying levels of *R. salmoninarum* infection (i.e., H-BKD and L-BKD groups) showed different transcript-dependent association on the PCO2 (Figure 11). Fish in the H-BKD group were closely associated with vectors including *mmp19, gvinp1, gzma, cldn1, lifr, socs1, ccl4, ifng, tnfsf14, irf1, camp*, and *herc6* as well as *R. salmoninarum 16S ribosomal RNA*. Nonetheless, other vectors such as *prkcd, cldn4, cfd, fcgr1, dock8, mmp13*, and *lmf2* showed more association with the L-BKD samples (Figure 11).

## Discussion

The present study is the first report on the transcriptome response of Atlantic salmon head kidney to BKD. In addition to transcriptome profiling of BKD responses in Atlantic salmon, we aimed to test if different *R. salmoninarum* levels influence the Atlantic salmon responses to this disease. All the fish in the present study were infected with the same dose of *R. salmoninarum*, but Taq-Man qPCR assays revealed a significant difference between the levels of infection in selected individuals with a higher (H-BKD) and lower (L-BKD) detected *R. salmoninarum* level at 13 dpi (Supplementary Figure S3). In this study, mortalities started at 24 dpi and 100% mortality occurred at 38 dpi (Supplementary Figure S1). Also, we did not find a significant correlation between fish weight and infection level at 13 dpi, as well as any difference in fish weight among the groups (i.e., control, L-BKD and H-BKD) at 13 dpi. Therefore, variations in the detected *R. salmoninarum* level of individuals can be attributed to the susceptibility, and the higher (H-BKD) and lower (L-BKD) infection level groups in the present study can also be considered as higher and lower susceptible groups, respectively. Using microarray analyses, we identified 7,729 and 6,766 DEPs in the L-BKD and H-BKD groups, respectively, compared with the control fish. The majority of the identified transcripts overlapped between groups (6,408 DEPs: 3,946 up- and 2,462 down-regulated DEPs). Also, there were 357 probes (171 up- and 186 down-regulated DEPs) responsive to the infection level (i.e., H-BKD vs. L-BKD). Our previous 44K microarray-based study identified 379 DEPs in the head kidney of Atlantic salmon injected with formalin-killed *R. salmoninarum* bacterin compared with the saline-injected control fish at 24 h post-injection (17). In addition, a previous SSH-based study identified 132 expressed sequence tags (ESTs) differentially expressed in response to *R. salmoninarum* infection in Chinook salmon at 24 and 72 h post-infection (hpi) (15). The larger number of transcripts identified herein compared to the previous *R. salmoninarum*-related studies may be attributed to the differences in response to live vs. killed pathogen, the studied time point (e.g., 1 vs. 13 days), species-dependent responses, and fish age (e.g., ~1,600 g fish in the bacterin study vs. ~54 g fish in the current study). In addition, following 14 days of infection, microarray (i.e., ~3.5K features) analysis identified 69 *P. salmonis-*responsive transcripts in the head kidney of Atlantic salmon (19). Using RNA-Seq, 825 and 412 transcripts were identified in Atlantic salmon spleen and head kidney, respectively, after 14 days *P. salmonis* infection (50). The differences in the numbers of responsive transcripts seen between the current and these previous studies may be caused by various factors such as experimental design, pathogen-dependent responses and the differences in transcriptome analyses (e.g., 3.5K vs. 44K microarray or RNA-Seq vs. microarray). The large number of *R. salmoninarum*-responsive transcripts compared to *P. salmonis*-responsive transcripts may also reflect the specific immune signalling triggered by different pathogen-associated molecular patterns (PAMPs) (e.g., Gram-positive vs. Gram-negative bacteria). The peptidoglycan of Gram-positive bacteria is recognised by TLR2, but TLR4 is the specific receptor for the lipopolysaccharides (LPS) of Gram-negative bacteria (51). Although they share some similarities, mammalian species were found to have distinct activated immune pathways in response to Gram-positive and Gram-negative bacteria (52, 53).

The present study used an IP challenge approach for profiling the transcriptome responses of Atlantic salmon to *R. salmoninarum* at 13 dpi. However, aspects such as route of infection, infection level, and patterns of disease spread in natural BKD outbreaks and saltwater environments may differ from the IP infection model used in the current study. Hence, *R. salmoninarum*-infected fish in aquaculture or natural environments may show some variations in dysregulation of the BKD-responsive biomarkers compared to the fish IP-challenged with *R. salmoninarum* in the present study. We studied the global gene expression response and discovered biomarkers for both lower and higher *R. salmoninarum* level/susceptible individuals, and the identified BKD-responsive biomarkers and pathways overlapping between lower and higher susceptible groups may represent the core immune response of Atlantic salmon to this pathogen. Nonetheless, further investigations involving Atlantic salmon naturally infected or bath challenged with *R. salmoninarum* in freshwater and seawater are needed to have a better grasp of the regulatory patterns of the identified biomarkers in the face of BKD.

We used samples with a higher (H-BKD; C_T_ values below 22) or a lower (L-BKD; C_T_ values above 25) level of infection (Supplementary Figure S3) to test if different levels of infection affect the BKD-dependent immune responses. Close clustering of samples in a given treatment based on the expression of all DEPs (Figure 2) suggests the comparable global gene expression responses of the samples associated with each group. Further, this reveals that the BKD-dependent response in Atlantic salmon head kidney can be influenced by the level of infection, and fish with different *R. salmoninarum* levels may have distinct transcriptional response patterns. There was a larger number of DEPs in the L-BKD group (7,729 DEPs) compared to the H-BKD group (6,766 DEPs), and 357 differentially expressed between the H-BKD and L-BKD groups. Since *R. salmoninarum* has immunomodulatory effects on its host (10), the infection level-dependent BKD responses of Atlantic salmon in this study may be related to host-pathogen interactions. Variations in the infection level of fish seen herein may also be influenced by individual-based differences in immune response, and/or specific immune signalling potentially modulated by *R. salmoninarum*. The infection level-responsive transcript list identified herein provides a more comprehensive understanding of the putative immune pathways affected by *R. salmoninarum*.

To validate the microarray results, we subjected 65 transcripts to qPCR analyses. These transcripts were from different comparisons (e.g., H-BKD vs. L-BKD), with different regulation (e.g., down- and up-regulated by BKD) and putative roles in various immune processes. All of the qPCR-studied transcripts, except for *fcrl5*, showed the same fold-change direction as the microarray experiment (Supplementary Table S4), although some microarray-identified transcripts (i.e., 3 out of 52 H-BKD vs. control and 14 out of 49 L-BKD vs. control differentially expressed transcripts) were not shown to have significant differential expression by qPCR. There were 11 microarray-identified transcripts responsive to *R. salmoninarum* infection level that were subjected to qPCR, 6 of which were confirmed by qPCR to have significant differential expression. On the other hand, we found significant differences in qPCR for some comparisons that did not show significant changes in the microarray study (Supplementary Table S4). The differences seen between microarray and qPCR results may be attributed to the variations in the distribution of acquired values (i.e., normalised fluorescence ratios vs. RQ values, respectively) as well as stringency level and statistical methods used for data analyses.

**Figure 12:**
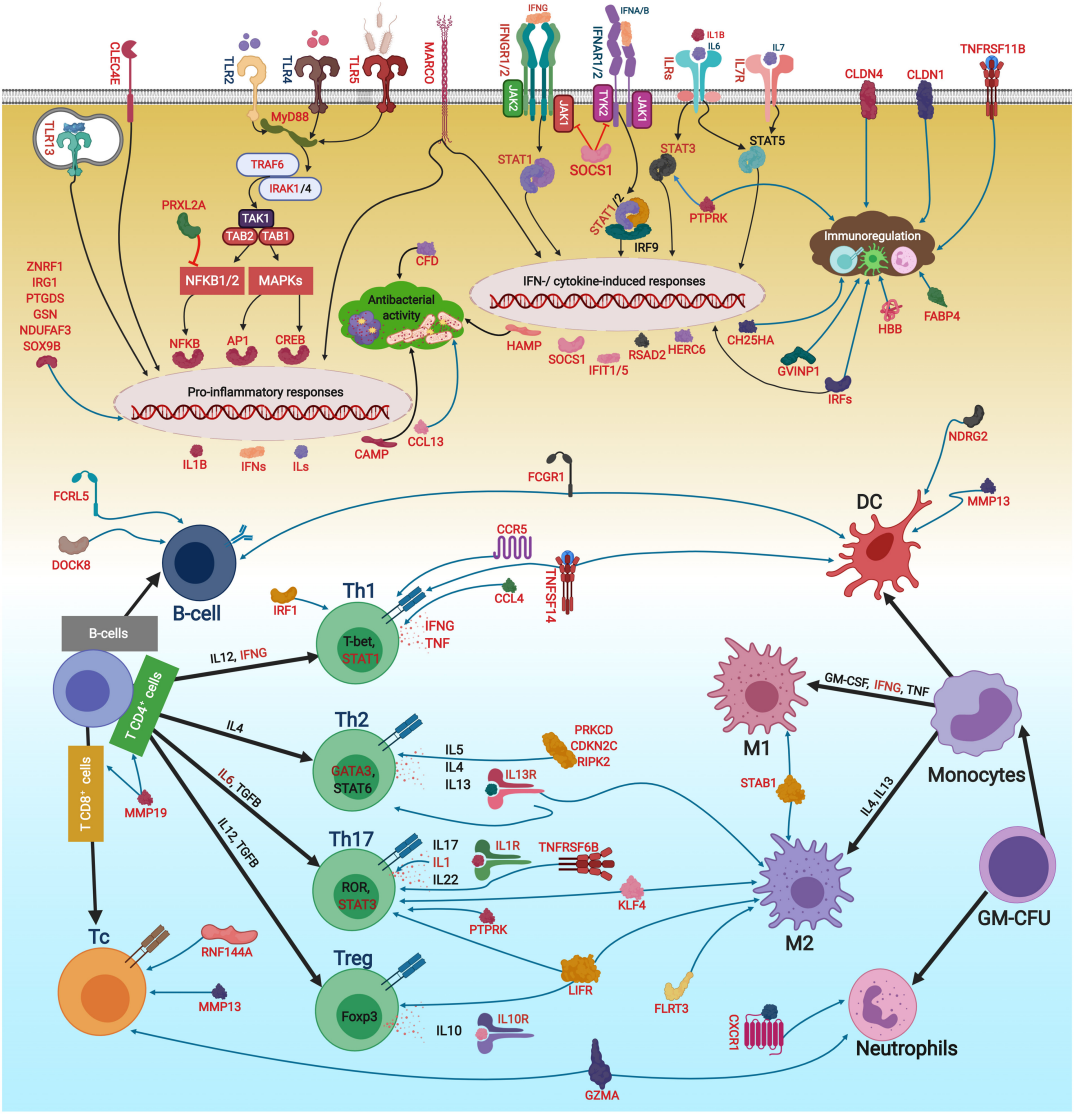
The putative adaptive and innate immune pathways differentially regulated by *R. salmoninarum* infection in Atlantic salmon head kidney. This figure was developed using the identified genes in this study and their known functions and regulatory pathways in mammals (54–58) as explained in the discussion section. This figure was created in BioRender (https://biorender.com/). The BKD-responsive genes are shown in red font or red boxes. The microarray results were not confirmed by qPCR for *sox9b* and *fcrl5*. Black and blue arrows show activatory and regulatory effects, respectively, whereas red lines indicate inhibitory effects. Oval circles and clouds reflect gene expression activation and biological processes, respectively. TLR, Toll-like receptor; MyD88, Myeloid differentiation primary response 88; TNF, Tumor necrosis factor; TRAF, TNF receptor-associated factor; IRAK, Interleukin-1 receptor-associated kinase; TAK1, Transforming growth factor beta, (TGFB)-activated kinase 1; TAB, TAK1-binding protein; NFKB, Nuclear factor kappa-B; MAPK, Mitogen-activated protein kinase; AP1, Transcription factor AP1; CREB, cAMP response element-binding protein; ILs, interleukins) IFNs, Interferons; IFNRs, IFN receptors; ILRs, IL receptors; JAK, Janus kinase; TYK, Tyrosine kinase; STAT, Signal transducer and activator of transcription; IRF, Interferon regulatory factor; RSAD2, Radical S-adenosyl methionine domain containing 2; CFD, Complement factor D precursor; HAMP, Hepcidin antimicrobial peptide; CCL13, C-C motif chemokine 13; HERC6, E3 ubiquitin-protein ligase herc6; CAMP, Cathelicidin antimicrobial peptide; CLDN1, Claudin-1; CCL,CC chemokine; STAB1, Stabilin-1; MARCO, Macrophage receptor MARCO; CLEC4E, C-type lectin domain family 4 member E; CH25HA, Cholesterol 25-hydroxylase-like protein A; TNFR11B, Tumor necrosis factor receptor superfamily member 11B; ZNRF1, E3 ubiquitin-protein ligase znrf; IRG1, Immune-responsive gene 1; CLDN4, Claudin 4; GVINP1, Interferon-induced very large GTPase 1; SOCS1, Suppressor of cytokine signalling 1; CXCR1, C-X-C chemokine receptor type 1; FABP4, Fatty acid-binding protein 4, adipocyte; GSN, Gelsolin; HBB, Haemoglobin subunit beta; NDUFAF3, NADH dehydrogenase, ubiquinone, 1 alpha subcomplex assembly factor 3; PRXL2A, Peroxiredoxin-like 2A; PTGDS, Prostaglandin D2 synthase, lipocalin; DOCK8, Dedicator of cytokinesis protein 8; TNFSF14, Tumor necrosis factor ligand superfamily member 14; LIFR, Leukemia inhibitory factor receptor; MMP19, Matrix metallopeptidase-19; CCL4, C-C motif chemokine 4; CDKN2C, Cyclin-dependent kinase inhibitor 2C; KLF4, Kruppel-like factor 4; CCR5,C-C chemokine receptor type 5; MMP13, Matrix metallopeptidase 13; DUSP7, Dual specificity protein phosphatase 7; RNF144A, E3 ubiquitin-protein ligase RNF144A-A; TNFR6B, Tumor necrosis factor receptor superfamily member 6B; GZMA, Granzyme A precursor; RIPK2, Receptor-interacting serine/threonine-protein kinase 2; PRKCD, Protein kinase C delta type; FCRL5, Fc receptor-like protein 5; PTPRK, Receptor-type tyrosine-protein phosphatase kappa-like; IL13RA1B, Interleukin 13 receptor alpha 1B; FCGR1, High affinity immunoglobulin gamma Fc receptor I; SOX9B, Transcription factor Sox-9-B; FLRT3, Leucine-rich repeat transmembrane protein FLRT3; NDRG2, N-myc downstream-regulated gene; GM-CSF, Granulocyte-macrophage colony-stimulating factor; TGFB, Transforming growth factor beta; T-Bet, T-cell-specific T-box transcription factor; GATA3, GATA binding protein 3; ROR, RAR-related orphan receptor gamma; FOXP3, forkhead box P3; GM-CFU, Granulocyte-macrophage colony forming units; DC, dendritic cell; M1/2, macrophage M1/2; Th1/2/17, T helper 1/2/17 cell; Treg, regulatory T cells.

The qPCR-studied transcripts were categorised based on their function in immune responses. Fourteen transcripts with putative roles in innate immune responses were included for qPCR validation (Figure 5). *R. salmoninarum* infection up-regulated the expression of the Atlantic salmon *tlr5, rsad2, cdf, hamp*, and *ccl* transcripts in both L-BKD and H-BKD groups compared with the control. While *ccl13* was only up-regulated in the L-BKD group, *casp14, herc6, camp*, and *cldn1* up-regulation was only seen in the H-BKD group. Further, *ccl, camp*, and *cldn1* were significantly up-regulated in the H-BKD compared with L-BKD group. Among innate immune relevant-studied transcripts, *cldn1* showed the strongest induction, i.e., 458-fold up-regulation in the H-BKD group compared with the control. *R. salmoninarum* infection down-regulated *stab1, marco*, and *clec4e* in both L-BKD and H-BKD groups, whereas *tlr13* down-regulation was only seen in the H-BKD group. Expression of *clec4e* negatively correlated with the infection level, as it was suppressed in the H-BKD group compared with the L-BKD fish. Our previous study (17) found the same fold-change direction in response to *R. salmoninarum* bacterin at 24 h post-injection for several of these transcripts (i.e., *tlr5, cdf, hamp, ccl, ccl13*, and *camp*), which indicates their importance in both early and late *R. salmoninarum*-related responses. Figure 12 depicts the innate and adaptive immune pathways activated by BDK in Atlantic salmon head kidney. Our results show the activation of innate immune responses downstream of TLRs. In parallel, the pathway enrichment analyses showed the dysregulation of BPs associated with Nuclear factor kappa-B (NFKB) activation, thereby increasing cytokine production (Figure 12). TLR5 of mammals and teleosts [e.g., rainbow trout and Japanese flounder (*Paralichthys olivaceus*)] is a pattern recognition receptor (PRR) that recognises bacterial flagellin, and activates the Myeloid differentiation primary response 88 (MyD88)-dependent pathway, resulting in the production of pro-inflammatory cytokines (59, 60). On the other hand, mammalian TLR13 recognises bacterial rRNA (61). Besides TLR13, we found down-regulation of transcripts encoding C-type lectin receptors, such as *clec4e* (alias *mincle*), which activates the inflammatory responses by recognising pathogenic fungi (62). Furthermore, we observed the BKD-suppressed expression of scavenger receptor-encoding transcripts, i.e., *stab1* and *marco*. In mammals, STAB1 is an essential factor for receptor-mediated endocytosis in macrophages (63), and MARCO is a PRR playing roles in bacterial binding and removal (64). In agreement with our results, Atlantic salmon *marco* was suppressed in *P. salmonis*-infected macrophages (19). MARCO was reported to bind to bacteria and be induced by *Vibrio anguillarum* in Ayu (*Plecoglossus altivelis*), suggesting a conserved bacterial binding function for teleost MARCO (65). The BKD-suppressed expression of PRR-encoding transcripts in the current study may be caused by the negative feedback loop triggered by immune responses or host immunomodulation exerted by *R. salmoninarum*.

The innate immune pathways activated by *R. salmoninarum* infection increased the expression of *hamp, camp, cdf*, and *ccl13*, which are associated with antibacterial processes (Figure 12). Teleost HAMP and CAMP [e.g., rainbow trout and European seabass (*Dicentrarchus labrax*)] exhibited antibacterial activities against both Gram-positive and Gram-negative bacteria (66–69). Mammalian CDF is involved in the alternative complement pathway (70). Human CCL13 showed antibacterial activity against *Pseudomonas aeruginosa* (71). Interestingly, we found a strong up-regulation of some antiviral biomarkers (i.e., *rsad2, herc6*, and *cldn1*) in response to BKD. Atlantic salmon *rsad2* and *herc6* were found to be responsive to a viral mimic in macrophage-like cells (28). Mammalian RSAD2 and HERC6 are known as IFN-induced proteins involved in antiviral processes (72, 73). Besides antiviral responses, *rsad2* induction by bacterial pathogens or LPS was reported in several species, including fishes (73, 74). However, the antibacterial response of *rsad2* may be species-dependent (47). Moreover, mammalian CLDN1 plays a role in antiviral responses as a co-receptor for viral entry (75), but its function in the fish immune response remains unknown. Although our qPCR, microarray and pathway enrichment results (e.g., over-representation of response to viruses) reflect the importance of factors, with putative roles in antiviral responses, in the Atlantic salmon response to BKD, further investigations are needed to determine the role of these transcripts in Atlantic salmon antibacterial responses. Considering the enrichment of IFN-related processes as well as the positive correlation between *rsad2* and *herc6* with the infection level (Supplementary Table S5), the up-regulation of antiviral biomarkers in the current study may be caused by secondary immune responses or immunomodulatory functions of *R. salmoninarum*. Furthermore, *R. salmoninarum* is an intracellular pathogen (9), and an intracellular bacterium (i.e., *Listeria monocytogenes*) was reported to induce mammalian IFN type I through IRF3-dependent signalling (76). Accordingly, stimulation of antiviral response-relevant transcripts by *R. salmoninarum* may be caused by activation of factors involved in the entry of intracellular pathogen into host cells or receptors recognising the bacterial DNA. BKD induction of *casp14* as an apoptotic or inflammatory caspase (77), alongside over-representation of BPs related to cell death in the current study, suggests the activation of apoptotic pathways following the inflammatory response. The identified transcripts involved in innate immune responses that showed positive (i.e., *camp, casp14, ccl, cldn1, herc6*, and *rsad2)* or negative (i.e., *marco* and *clec4e*) correlation with *R. salmoninarum* level (Supplementary Table S5) can be suggested as suitable biomarkers for assessing the infection level-dependent responses of Atlantic salmon to BKD.

BKD influenced molecular pathways (e.g., regulation of leukocyte migration, negative regulation of immune response, regulation of NLRP3, inflammasome complex assembly and regulation of cytokine biosynthetic process) associated with regulation of immune responses, and several qPCR-studied transcripts in our study play putative immunoregulatory roles (Figure 12). For example, *R. salmoninarum* induced *ch25ha, tnfrsf11b, znrf1*, and *socs1* in both infection levels, whereas BKD induction of *cldn4, irg1*, and *gvinp1* occurred in either the L-BKD or the H-BKD group (Figure 6). IFN-induced CH25H shows antiviral activities and can positively and negatively regulate the inflammatory responses of mammals (78). Mammalian TNFRSF11B (alias Osteoprotegerin), ZNRF1 and CLDN4 regulate LPS-induced cytokine and/or inflammatory responses (79–81). GVINP1 is an IFN-induced GTPase, regulating oxidative and inflammasome-related antimicrobial activities of cells in mammals (82). Cytokine-activated IRG1 of zebrafish (*Danio rerio*) links cellular metabolism with immune defence through regulating mitochondrial reactive oxygen species (ROS) production (83). Moreover, *irg1* was up-regulated in *P. salmonis*-infected Atlantic salmon head kidney (19). The expression patterns of *irg1* and *gvinp1* were comparable with *socs1*, suggesting that these transcripts may share an activation pathway. Cytokine-inducible mammalian SOCS1 inhibits JAK/STAT signalling by binding to JAKs downstream of the cytokine receptors (Figure 12) (84). Significantly higher induction of *socs1* in the H-BKD group and its positive correlation with *R. salmoninarum* level (Supplementary Table S5) suggest this transcript as an important biomarker for infection level-dependent responses of Atlantic salmon to BKD. In agreement with *socs1* results, the JAK-STAT-related signalling pathway was found to be dysregulated in response to *R. salmoninarum* level. Taken together, the infection level-dependent response of *socs1* and other putative IFN-/Cytokine-inducible transcripts (*irg1* and *gvinp*), as well as pathway enrichment results, suggest that the JAK-STAT pathway may be an essential part of host-pathogen interactions between Atlantic salmon and *R. salmoninarum* (Figure 12).

In addition to these up-regulated transcripts, our qPCR results showed suppression of transcripts with putative immunoregulatory functions. *R. salmoninarum* infection down-regulated *cxcr1, fabp4, gsn, ndufaf3, prxl2a, ptgds*, and *hbb* in both H-BKD and L-BKD groups (Figure 6). Mammalian CXCR1 recruits neutrophils to the inflammation site and regulates their bactericidal activity (85). The same fold-change direction was seen for *cxcr1* in LPS-exposed peripheral blood leucocytes of fugu (*Takifugu rubripes*) (86) and *R. salmoninarum* bacterin-injected Atlantic salmon (17). Besides its fatty acid-related function, FABP4 of mammals regulates the Inhibitor of nuclear factor kappa-B kinase (IKK) signalling pathway that activates the production of inflammatory cytokines (87). A previous study from our group found Atlantic salmon *fabp4* as a dietary fatty acid-responsive transcript (28). We found intracellular lipid transport process to be differentially regulated with BKD; further studies are needed to determine the association of Atlantic salmon antibacterial responses with *fabp4* and other lipid metabolism-relevant (e.g., *ch25ha)* transcripts identified herein. We also identified BKD-responsive transcripts involved in the regulation of inflammation. Recombinant GSN of mammals has been shown to inhibit LPS-induced cytokine responses (88). Mammalian NDUFAF3 is a factor associated with Mitochondrial Respiratory Complex I, which was found to modulate LPS-induced NFKB activation and pro-inflammatory responses (89). Similarly, PRXL2A inhibits Mitogen-activated protein kinase (MAPK) and NFKB signalling pathways in humans (90). Prostaglandins play immunoregulatory roles in teleosts (91), and PTGDS (aliases PGD2) was found to suppress *V. anguillarum* DNA-induced expression of *il1b* in gilthead seabream (*Sparus aurata*) granulocytes (92). *P. salmonis* repressed the expression of *ptgds* in Atlantic salmon head kidney (19). Considering that *P. salmonis* is an intracellular Gram-negative pathogen, the same gene expression regulation (e.g., *ptgds* suppression and *irg1* induction) of Atlantic salmon in response to *P. salmonis* (19) and *R. salmoninarum* may be linked to a common molecular pathway activated by intracellular bacteria. The suppressed expression of genes encoding putative immune modulators identified in the present study may be attributed to the host's need for boosting pro-inflammatory responses. Furthermore, haemoglobin-derived peptides of human and fish show antibacterial activities (93, 94), and HBB was reported to regulate the antiviral innate immune responses in humans (95). Additional studies are needed to develop a better understanding of the immunoregulatory functions of teleost *hbb* and other BKD-suppressed transcripts identified in this study.

There were several pathways (e.g., myeloid cell differentiation, regulation of stem cell differentiation, positive regulation of interleukin-12 production and interleukin-12 secretion) involved in the regulation of lymphocyte differentiation that were dysregulated in response to *R. salmoninarum* infection (Figures 3, 12). Our qPCR results showed up-regulation of *il1b, irf1, dock8*, and *ccl4* in both BKD groups, whereas *ifng, mmp19*, and *lifr* induction was only significant in the H-BKD group (Figure 7). *R. salmoninarum* infection suppressed *cdkn2c, klf4*, and *ccr5* expression in both BKD groups, although *il7r* down-regulation only happened in the L-BKD group. These transcripts, except for *dock8*, are known to play roles in mammalian T-cell differentiation. DOCK8 is a crucial effector involved in TLR9-driven differentiation of B-cells (96). The pathway activated by IL1B provides a pro-survival signal for T-cells and triggers the differentiation of T helper 17 (Th17) cells from naïve T-cells (97). Besides its diverse immunoregulatory roles, IFNG is a necessary cytokine for differentiation of the naïve CD4^+^ T-cells into Th1 cells (97). In addition to IL12, IFNG and STAT4, IRF1 is required for the differentiation of naïve T-cells into Th1, but not Th17 cells (98). MMP19 was also found to perform an essential function in T-cell differentiation (99). LIFR plays regulatory roles in the differentiation of the Th17 and regulatory T (Treg) cells (100). Our findings suggest the crucial role of Treg and Th differentiation-related processes in Atlantic salmon defence mechanisms against *R. salmoninarum*. In mammalian species, CCL4 is chiefly expressed by APCs and B-cells, and it can engage with CCR5 expressed on IFNG-producing Th1, thereby regulating polarisation and trafficking of Th1 cells (101). LPS-induced CCL4 of orange-spotted grouper (*Epinephelus coioides*) was reported to attract leukocytes and stimulate the Th1 differentiation pathway (102), suggesting the conserved function of mammalian and teleost CCL4. The opposite regulations of *ccl4* and *ccr5* in our study may be caused by negative feedback loops, or suggest that they can be regulated through different immune pathways. IL7R mediates T-cell and B-cell differentiation through activation of JAK1/3 and consequently STAT5 (103, 104). Besides its role in macrophage differentiation, KLF4 is a transcription factor required for Th17-cell differentiation and IL17 production (105). CDKN2C was found as an important factor associated with induction of GATA3-dependent Th2 cell proliferation (106). In agreement with *cdkn2c* expression, our microarray data showed down-regulation of Atlantic salmon *gata3* in response to *R. salmoninarum* infection, indicating the putative conserved roles of these genes in Atlantic salmon and mammals. Among identified lymphocyte differentiation biomarkers, *ccl4, mmp19*, and *lifr* showed *R. salmoninarum* level-dependent responses, suggesting them as suitable biomarkers assessing the Atlantic salmon response to BKD intensity. While our results suggested the putative role of these transcripts in antibacterial responses of Atlantic salmon, further studies are needed to functionally characterise these transcripts and their encoded proteins.

Our microarray data showed BKD-dependent dysregulation of pathways (e.g., T-cell activation, positive regulation of lymphocyte activation and lymphocyte chemotaxis) related to lymphocyte function (Figures 3, 12). As shown by qPCR, *mmp13* was up-regulated in both BKD groups. Mammalian MMP13 is a Tumor necrosis factor alpha (TNFA)-induced protein mediating the conversion of the inactive form of Transforming growth factor beta 1 (TGFB1) to the active form, which facilitates TLR-driven immunoglobulin switching in B-cells (107). *R. salmoninarum* infection up-regulated *prkcd* only in the L-BKD group, whereas *dusp7, rnf144a, tnfrsf6b, gzma*, and *ripk2* were only significantly induced in the H-BKD group (Figure 8). Mammalian PRKCD plays a role in the up-regulation of IL10 (108). Mouse RIPK2 is a kinase involved in TLR2-activated IL10 production in response to Gram-positive *Streptococcus pneumoniae* (109). DUSPs may manage MAPK activation via negative feedback loops (110), and human DUSP7 was suggested to have an effector function associated with Th1 (111). Mammalian TNFRSF6B (alias DcR3) is an immunoregulator for Th17 cell activity and cytokine responses (112), and our previous study showed its transcriptional induction in Atlantic salmon stimulated with *R. salmoninarum* bacterin (17). In the current study, qPCR data showed the up-regulation of transcripts with putative roles in cytotoxic T-cell function. For example, *gzma* showed a *R. salmoninarum* level-dependent response in Atlantic salmon. Granzymes are cytolytic granules involved in the cell-mediated cytotoxic activity of CD8^+^ T-cells (113). GZMA was revealed as the main granzyme playing a role in cell-mediated cytotoxicity of teleosts (gilthead seabream and European seabass) (114). In addition, mammalian RNF144A is a key factor balancing IL2R-dependent responses of CD8^+^ T-cells, thereby preventing severe inflammation (115). Although up-regulation of teleost [i.e., grass carp (*Ctenopharyngodon idellus*)] *rnf144a* in response to viral infection was previously reported (116), further studies are needed to characterise the function of this and other identified transcripts in fish antibacterial responses. In the current study, *ptprk, tcra*, and *il13ra1b* were suppressed in Atlantic salmon head kidney in response to *R. salmoninarum* infection. As reported in mammalian species (117), the BKD-suppressed response of *tcra* may be attributed to the negative feedback mechanism for managing pathogen elimination and minimising the inflammatory damages. PTPRK contributes to T-cell pathogenesis through STAT3 dephosphorylation, in which its under-expression results in STAT3 activation (118). As found by microarray, the slight up-regulation of STAT3 in our study may be associated with PTPRK regulatory function, but further studies are needed to determine if this function is conserved in fishes. IL13RA1 is a receptor expressed on various immune cells such as B-cells. Engagement of IL13RA1 with IL13, a regulatory cytokine produced by Th2 cells, elicits multiple immune processes such as STAT6 activation and promotion of IgE production in B-cells (119). Infection level-dependent dysregulation of *il13ra1b* and other identified lymphocyte-associated biomarkers (i.e., *prkcd, gzma*, and *ripk2*) suggests the importance of T-cell activated pathways in interactions between Atlantic salmon and *R. salmoninarum* (Figure 12).

**Figure 13:**
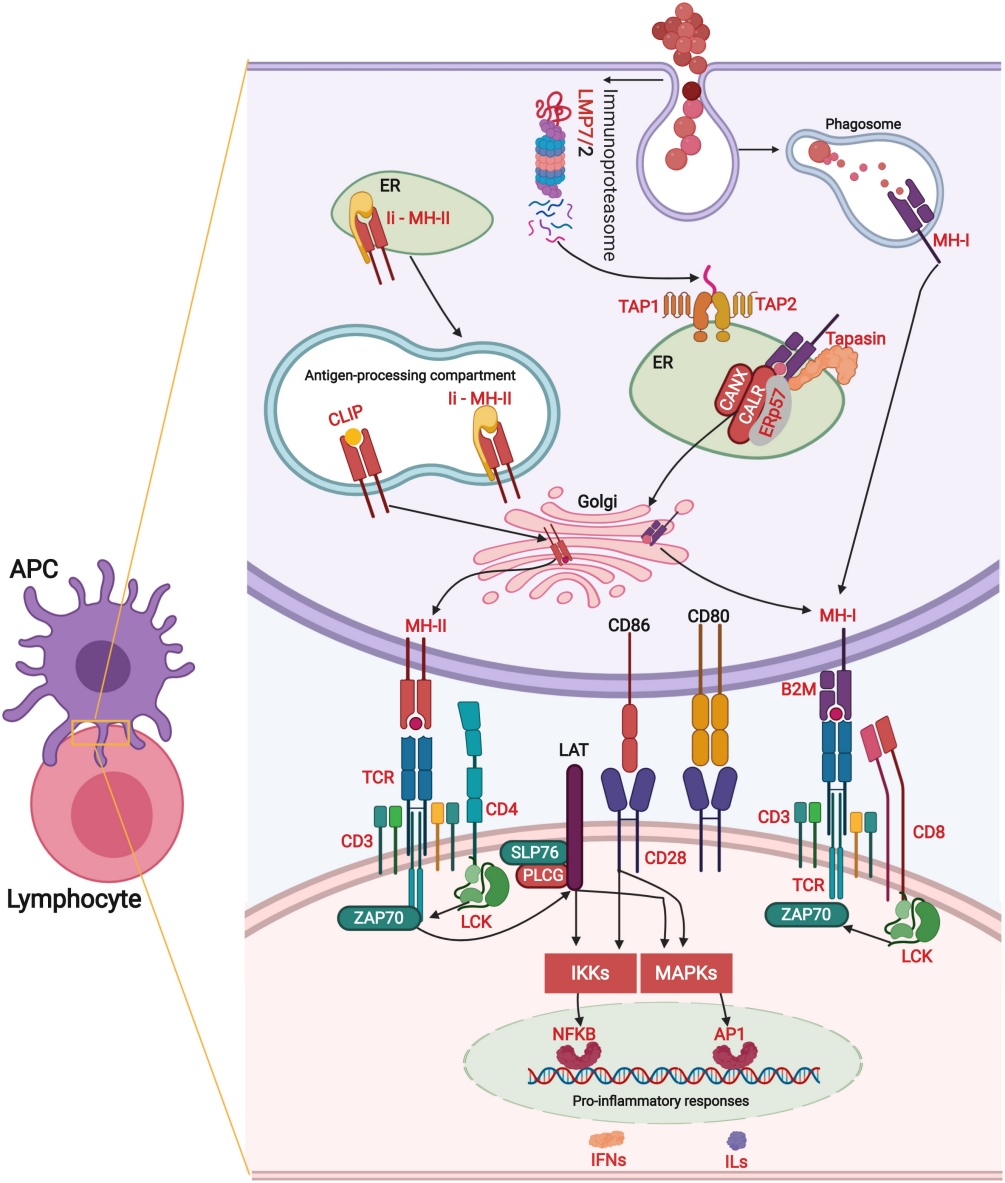
The antigen-processing and presenting pathways differentially regulated by *R. salmoninarum* infection in Atlantic salmon head kidney. This figure was developed using the identified genes in this study and their known functions and regulatory pathways in mammals as explained in the discussion section (54, 120–122). This illustration was generated using BioRender (https://biorender.com/). The BKD-responsive genes are shown in red font or red boxes. The microarray results were not confirmed by qPCR for *mh1*. Black arrows show activatory effects. Oval circles reflect gene expression activation. APC, antigen-presenting cell; ER, endoplasmic reticulum; LMP2/7, Immunoproteasome subunits LMP2/7; Ii, Invariant chain Ii or CD74; MH-I/II, [Major histocompatibility I/II, known as Major histocompatibility complex I/II(MHC-I/II) in mammals]; CLIP, class II-associated invariant chain peptide; TAP, Protein associated with antigen processing; Tapasin, TAP-associated glycoprotein; ERp57, Endoplasmic reticulum protein of 57 KDa; CANX, Calnexin; CALR, Calreticulin; CD, Cluster of differentiation; B2M, Beta-2 microglobulin; TCR, T-cell receptor; SLP76, SH2 domain containing leukocyte protein of 76kd; LAT, Linker for activation of T-cells; PLCG, Phospholipase gamma; LCK, Lymphocyte -specific tyrosine kinase; ZAP70, Tyrosine kinase zeta chain-associated protein of 70 kD; IKK, NFKB1 inhibitor kinase; NFKB, Nuclear factor kappa-B; MAPK, Mitogen-activated protein kinase; AP1, Transcription factor AP1; ILs, Interleukins; IFNs, Interferons.

APC-related processes (e.g., antigen processing and presentation of peptide antigen via MHC class I and antigen processing and presentation of exogenous antigen) were found to be dysregulated in *R. salmoninarum*-infected Atlantic salmon (Figure 3). As shown in Figure 13, our transcriptome profiling identified several *R. salmoninarum*-responsive transcripts involved in antigen-presenting and processing pathways. The teleost MH-I-dependent pathway exhibits several differences with their mammalian counterparts (123–128); for example, different paralogues (i.e., Calnexin) and splice variants (i.e., Tapasin) of molecules involved in the MH-I pathway were identified and suggested to play diverged or unique roles in salmonid antigen presentation (123–128). The qPCR results showed the *R. salmoninarum*-dependent up-regulation of *fcgr1* and *tnfsf14*, as well as down-regulation of *cdh1, flrt3*, and *ndrg2* (Figure 9). FCGR1 is a member of the Fc receptor protein family that links adaptive and innate immune responses through regulation of antibody activity and modulation of dendritic cell (DC) functions (e.g., antigen presentation and phagocytosis) (129). Mammalian TNFSF14 plays an essential role in the induction of DC maturation (130). CDH1 was described to be involved in cell-cell adhesion processes such as T-cell and DC interactions (131). Also, *flrt3* and *cdh1* were suggested to be associated with mammalian macrophage function, as they were strongly up-regulated in IL10-induced M2 macrophages and IL-4 + IL-10-stimulated bone marrow-derived macrophages, respectively (132). NDRG2 modulates the activation and differentiation of DCs as well as DC-mediated T-cell activation (133). The BKD-dependent induction of *mh1* was not confirmed by the qPCR, although our transcriptome data showed the activation of several co-receptors and immune effectors involved in antigen presentation pathways, reflecting the importance of MH-dependent pathways in Atlantic salmon response to BKD (Figure 13). Many pathogens employ strategies to evade MHC pathways (134). While bacterial immunoevasion of the MHC pathways were mainly associated with class II molecules, intracellular bacteria such as *Mycobacterium tuberculosis* and *Listeria monocytogenes* were found to suppress the expression of mammalian *mhc1* (134, 135). It remains unknown if *R. salmoninarum* possesses a mechanism for evading the antigen presentation pathway. Considering immunosuppressive features of *R. salmoninarum* (10), further studies are required to determine the interaction of this intracellular pathogen with the teleost MH-I pathway, test if its immunomodulatory features are related to MH-I molecule, and characterise the APC function-relevant transcripts identified herein.

*R. salmoninarum* induced the expression of *rtp2, rtp3, lmf2*, and *mbip*, and suppressed the expression of *dscam, tmod4*, and *cpxm2* in both H-BKD and L-BKD groups (Figure 10). In addition, there was an up-regulation of *gda* in the H-BKD group compared with both the L-BKD and control groups. The immune roles of these identified BKD-responsive transcripts in mammals remain undescribed. For example, while RTPs are established as odorant receptors in mammals (136), it is not known if mammalian RTPs play immune-relevant roles. GDA and TMOD4 are involved in the regulation of the neural response (137) and structural development (138), respectively. DSCAM is involved in the immune response of arthropods (139), and was found to be associated with zebrafish development (140). Further studies are needed to describe the immune function of vertebrate DSCAM. Nonetheless, since *dscam* suppression was correlated with a higher level of infection, our results suggest *dscam* as a suitable biomarker transcript for assessing the *R. salmoninarum* infection level-dependent response of Atlantic salmon.

In summary, the present study profiled the transcriptome of Atlantic salmon head kidney in response to different levels of *R. salmoninarum* infection. We developed a more complete picture of the genes and molecular pathways underlying the Atlantic salmon immune response to BKD. *R. salmoninarum* infection dysregulated transcripts encoding PRRs (e.g., *tlr5*), signal transducers (e.g., *traf6*), and transcription factors (e.g., *nfkb1*) associated with the MyD88-dependent pathway, resulting in activation of cytokines (e.g., *ifn*s and *il*s) and antimicrobial factors (e.g., *camp* and *hamp*). BKD also activated cytokine-dependent responses (e.g., JAK-STAT mediated pathway and IFN-induced transcripts) and immune regulators (e.g., *znrf1* and *irg1*). Furthermore, a large number of transcripts associated with adaptive immune responses such as T-/B-cell differentiation (e.g., *irf1, dock8*, and *ccl4*) and function (e.g., *rnf144a* and *tnfrsf6b*) as well as antigen presentation (e.g., *fcgr1* and *tnfsf14*), were identified as BKD-responsive in this study. The JAK-STAT signalling pathway was found to be influenced by *R. salmoninarum* level, suggesting the importance of IFN-dependent pathways in host-pathogen interactions. PCoA showed the significant correlation of BKD-responsive transcripts with a higher (e.g., *mmp19, gvinp1, cldn1, lifr, socs1, ccl4*, and *ccl*) or lower (e.g., *prkcd, cldn4*, and *cfd*) level of *R. salmoninarum*. These transcripts can be used as biomarkers to assess the infection level-dependent BKD responses of Atlantic salmon, and are suitable candidates for investigating the host component of interactions between Atlantic salmon and *R. salmoninarum*. We identified and qPCR validated *R. salmoninarum*- and infection level-responsive biomarker transcripts, which are valuable tools for future research (e.g., development of therapeutic diets and vaccines) for improving the Atlantic salmon resistance to BKD. Further studies characterising the functions of transcripts identified herein can enhance the current understanding of molecular processes involved in BKD-related responses of Atlantic salmon.

## Data Availability Statement

The datasets generated for this study can be found in online repositories. The names of the repository/repositories and accession number(s) can be found at: https://www.ncbi.nlm.nih.gov/geo/, GSE150335.

## Ethics Statement

All procedures in the present study were performed based upon the guidelines of the Canadian Council on Animal Care.

## Author Contributions

KE took a lead role in TaqMan assays, sample selection, microarray experimental design, microarray analyses, qPCR validation design, data analyses, data interpretation, and the writing of the manuscript draft. AC-S helped with enrichment analyses and writing a part of the manuscript. SMI helped with primer design and carried out the qPCR assays. ME helped with TaqMan assays and PCoA analysis. SK assisted with the annotation of the microarray results. CB helped with sampling and RNA extraction of the samples. RA-H was involved in the experimental design, bacterial culture and infection challenge, and reviewed the manuscript. EJ managed the experimental design, infection trial and samplings, and took a part in manuscript preparation. MLR was involved in experimental design, sample selection, microarray experimental design, data analyses and data interpretation, and took an active role in manuscript writing. All authors read and approved the final manuscript.

## Conflict of Interest

EJ and CB are employed by Cargill Aqua Nutrition. EJ, in the representation of Cargill Innovation, designed the fish infection trial and oversaw the fish trial execution. CB was the lead technician for the experimental fish trial as well as RNA extraction. The remaining authors declare that the research was conducted in the absence of any commercial or financial relationships that could be construed as a potential conflict of interest. The handling editor declared a past co-authorship with several of the authors SI, KE, and MR.
