# Supplementary material for: Transcriptomic Profiling of the Adaptive and Innate Immune Responses of Atlantic Salmon to Renibacterium salmoninarum Infection
Source: Front Immunol. 2020 Oct 28;11:567838. doi: 10.3389/fimmu.2020.567838 (PMC7656060; doi:10.3389/fimmu.2020.567838)
Supplement: Supplementary file 1 [file Presentation_1.PDF]

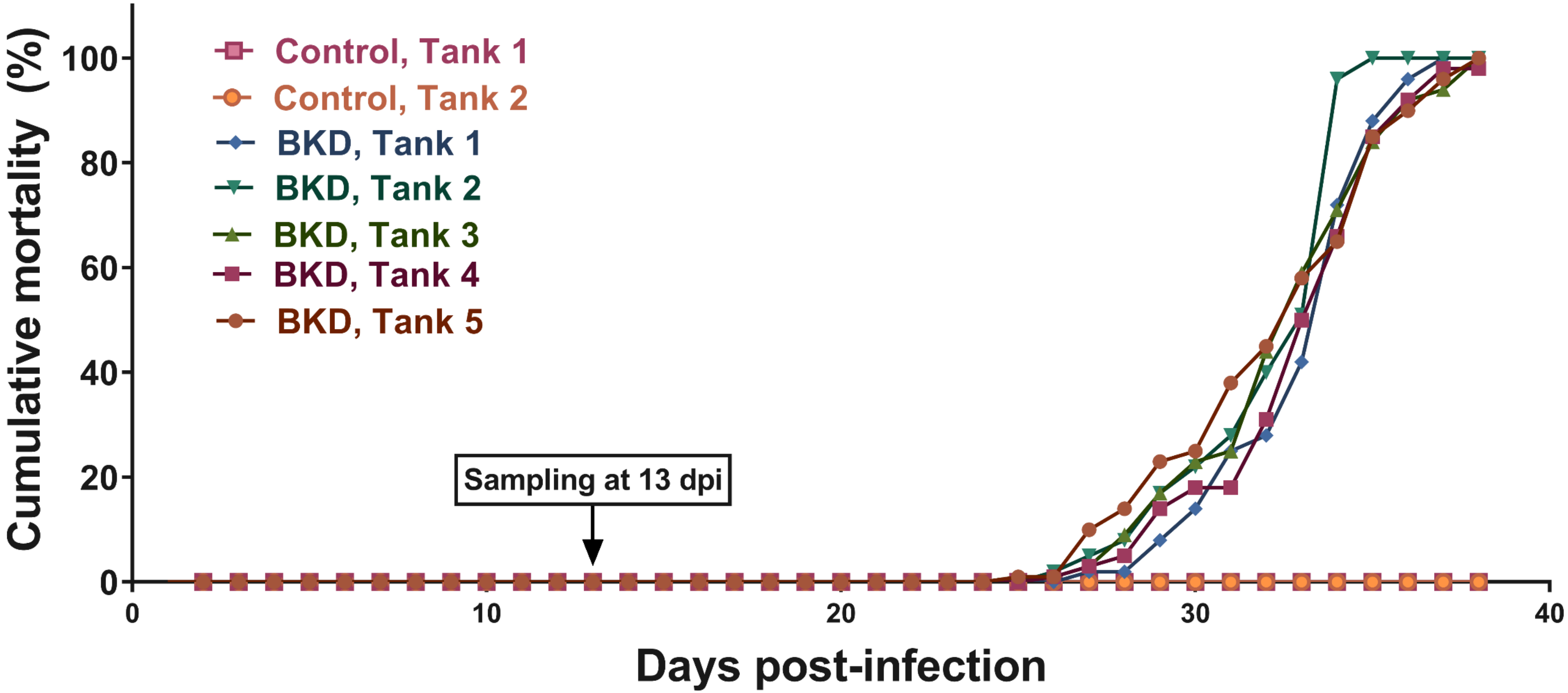

Supplemental Figure S1.

Cumulative mortality of Atlantic salmon in different tanks used for the current study. Fish in the control tanks were injected with sterile KDM-2 medium, whereas fish in the BKD tanks were injected with *R. salmoninarum* (final concentration of 2×10^8 CFU per fish). Tanks 1 and 2 in each group were used for sampling the fish head kidney at 13 days post-infection/injection (dpi), and tanks 3, 4 and 5 in the BKD group were only used for monitoring the mortality.

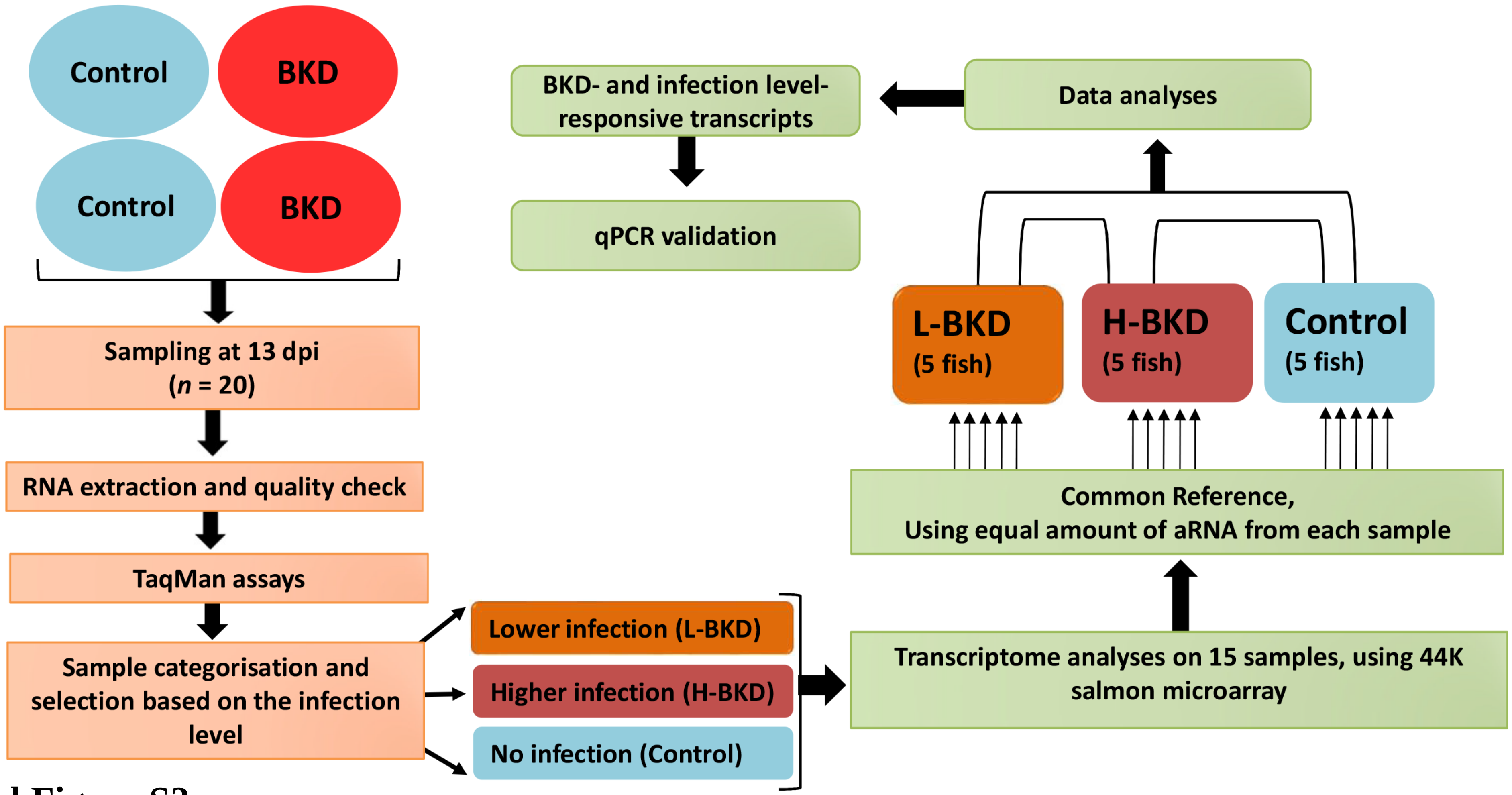

Supplemental Figure S2.

Overview of experimental design. Atlantic salmon in experimental tanks (i.e. 2 sampling tanks per treatment) were injected with 2×10^8 colony forming units (CFU) of *R. salmoninarum* (BKD) or sterile KDM-2 medium (Control). The head kidneys of infected and control fish were collected (10 fish per tank) at 13 days post-infection/injection (dpi). Using RNA-based TaqMan assays for the expression of *R. salmoninarum 16S ribosomal RNA* and Atlantic salmon *elongation factor 1 alpha-1* (*ef1a1*), the infection level of samples was determined, and samples with higher (H-BKD) and lower (L-BKD) levels of *R. salmoninarum* infection as well as control group (i.e. no infection) were used for the transcriptome analyses.

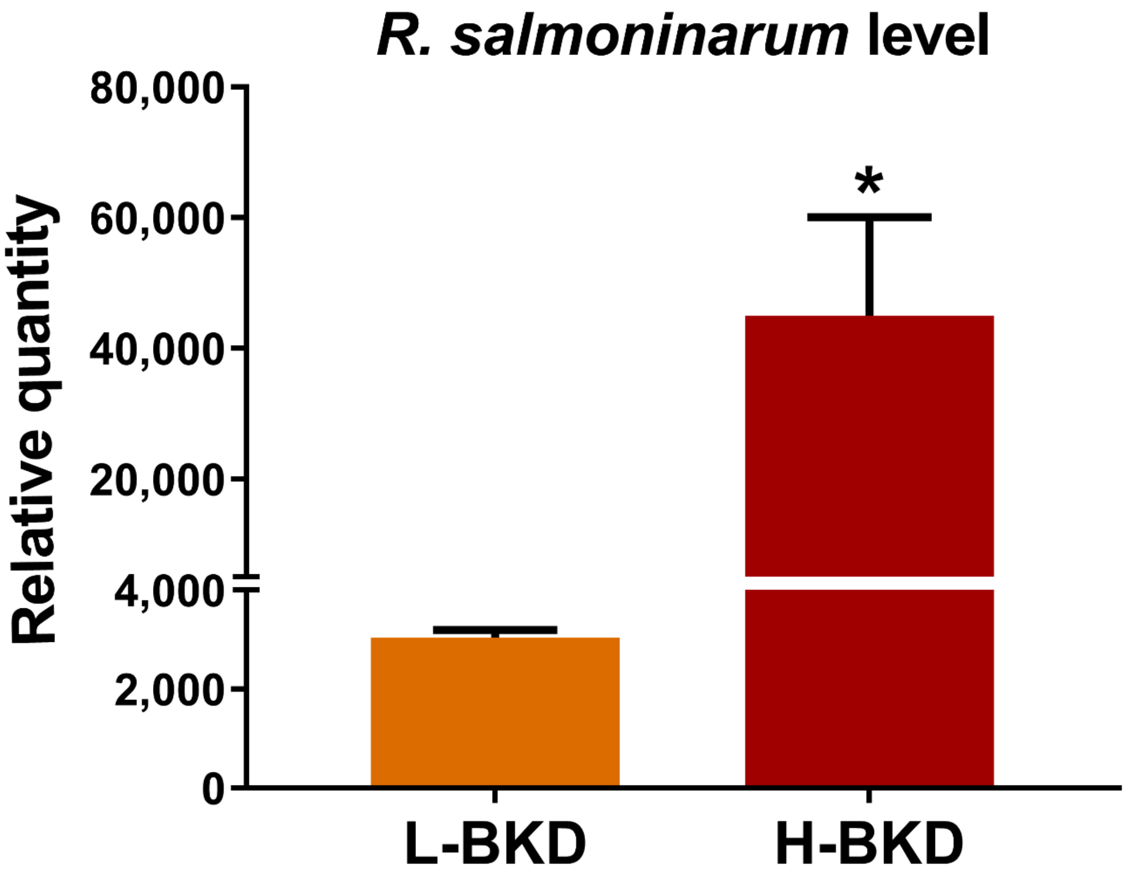

Supplemental Figure S3.

Levels of *R. salmoninarum* infection in Atlantic salmon head kidney, as determined by TaqMan assays. The expression of *R. salmoninarum 16S ribosomal RNA* normalised to Atlantic salmon *elongation factor 1 alpha-1* (*ef1a1*) indicates the infection level. Data are presented as mean ± SE ($n = 5$). All fish were injected with the same dose of live *R. salmoninarum*, but they showed different levels of infection at 13 dpi, as found by RNA-based TaqMan assays. Fish with a higher level of infection were categorised as H-BKD, whereas samples showing a lower level of infection were included in the L-BKD group. Level of *R. salmoninarum* infection significantly varied between the H-BKD and L-BKD groups (unpaired t-test; $p < 0.05$).
