# Supplementary material for: Transcriptomic Profiling of the Adaptive and Innate Immune Responses of Atlantic Salmon to Renibacterium salmoninarum Infection
Source: Front Immunol. 2020 Oct 28;11:567838. doi: 10.3389/fimmu.2020.567838 (PMC7656060; doi:10.3389/fimmu.2020.567838)
Supplement: Supplementary file 2 [file Table_1.PDF]

Supplemental Table S1. Primers used in qPCR studies

Name and symbol	GenBank accession number		Primer sequence 5' to 3'	R^2	Amplification efficiency (%)	Amplicon size (bp)
**Innate immune response transcripts**						
toll-like receptor 5 (tlr5)[b]	AY628755	Forward	ATCGCCCTGCAGATTTTATG	0.997	98.0	103
		Reverse	GAGCCCTCAGCGAGTTAAAG			
radical s-adenosyl methionine domain containing (*rsad2*)[a]; alias *viperin*	BT047610	Forward	ACCATTTTACCCGACAGTGC	0.994	94.2	183
		Reverse	TCCCCAAGAAATCACCTCTG			
complement factor D precursor (*cfd*)[b]	BT058155	Forward	ATCCGCTCAGTGGTTCTTCA	0.994	101.7	147
		Reverse	TTGACCTCGGGACTGTAAGG			
hepcidin antimicrobial peptide (*hamp*)[b]	BT125319	Forward	ATGAATCTGCCGATGCATTTC	0.996	88.9	134
		Reverse	AATGGCTTTAGTGCTGGCAG			
C-C motif chemokine 13 (*ccl13*)[b]	BT048088	Forward	ACTCCTCCTGGGACTGCTCT	0.994	100.0[h]	109
		Reverse	CCTCTTTGGGTGGAACTTCA			
caspase-14 (*casp14*)	BT048360	Forward	GAAACCCAAGCTCTTCGTTG	0.995	107.6[h]	175
		Reverse	CGGGTGTATAATCGCCAGTT			
E3 ubiquitin-protein ligase herc6 (*herc6*)[a]	EG915319	Forward	CACCGGCTAACAGTGATGTG	0.996	99.0[h]	128
		Reverse	GCACCCAGTTGTCTGAAGGT			
cathelicidin antimicrobial peptide (*camp*)[b]	GQ870278	Forward	AAGCCAGAAAATGCTCCAGA	0.992	103.8	107
		Reverse	ACCCTCAGGACGACCAATTA			
claudin-1 (*cldn1*)	DY697380	Forward	GCTCTTGCTTTGATCGGTCT	0.996	98.4	123
		Reverse	TGACATCCATAACCCCAGGT			
CC chemokine (*ccl*)[b]	EG850594	Forward	TTCCCTGTGTCAATGCTGTC	0.995	96.7	137
		Reverse	GGTGGTGTTCTGTGTGTCCA			
stabilin-1 (*stab1*)	GO049975	Forward	TCCCCCTCCTCTTCATTCTT	0.996	102.8	114
		Reverse	CATCCTGGTCACCTGTCTGA			
macrophage receptor with collagenous structure (*marco*)	AJ425462	Forward	GCACAGGCACAGAATCCAG	0.994	97.2	153
		Reverse	TCCTCCAGTCTTTTCGATGG			
toll-like receptor 13 (*tlr13*)	GO064286	Forward	TGACAGCCATGAGGAGTGAG	0.996	103.0	124
		Reverse	TCACCCTGGTAGCATCCTTT			
c-type lectin domain family 4 member e (*clec4e*)[b]	EG928463	Forward	CCACCAATCACGCAACAT	0.984	104.3	115
		Reverse	TCACGCCTCTTCACTTCTCA			
**Innate immune regulatory transcripts**						
cholesterol 25-hydroxylase-like protein a (*ch25ha*)[b]	BT046542	Forward	TAGAGCTGTGATGCTAGTTTAC	0.994	97.3	106
		Reverse	ACCCAGTAGCACTGAGAAGTC			
tumor necrosis factor receptor superfamily member 11B (*tnfrsf11b*)[b]	BT049358	Forward	CTGTCCTCAGGGGTACGTGT	0.998	93.7[h]	154
		Reverse	CTGACCAGCTTCCTCAGCTT			
E3 ubiquitin-protein ligase znrf1 (*znrf1*)[b]	EG922586	Forward	CAGCACGTCATCGTTGTAGG	0.994	90.3[h]	103
		Reverse	CAAGTGTCCTGTCTGCTCCA			
immune-responsive gene 1 (*irg1*); alias *aconitate decarboxylase 1* (*acod1*)	DW584044	Forward	CTCAACGCAGTTCACCTGAC	0.998	93.6	181
		Reverse	GCAGTGTCATCCCTGATCCT			
claudin 4 (*cldn4*)[b]	BT048350	Forward	CAACATCGTGACTGCTCAGG	0.996	97.7	108
		Reverse	GGTAAGGCCAGAAGGGAGTC			
interferon-induced very large GTPase 1 (*gvinp1*)	EG826043	Forward	GGTGCCACTCATTCTGACAA	0.994	96.3	185
		Reverse	AATCTGGCAATACCCAATGC			
suppressor of cytokine signalling 1 (*socs1*)[a]	EG924375	Forward	CTGTAGGATGGTCGCTCACA	0.999	96.3	133
		Reverse	ACACTGTTTGGATGGGTGCT			
C-X-C chemokine receptor type 1 (*cxcr1*)[b]	CX355704	Forward	ATGCTGATTCCCCCTACTCC	0.995	104.0[h]	103
		Reverse	ACACTGCTCAAGCCCAAGAT			
fatty acid-binding protein 4, adipocyte (*fabp4*)[a]	NM_001141203	Forward	GACTTGGGACGGCAAGACTA	0.997	105.0	128
		Reverse	CAGCAGACTGGAATCACACC			
gelsolin (*gsn*)	EG922584	Forward	CATGGCTTCTTCTCCAGCAT	0.990	104.4	103
		Reverse	TCTGGCTTATGGGTCCAACT			
haemoglobin subunit beta (*hbb*)	GO061840	Forward	GTTGCCGAAGGTGCTAAAGT	0.997	85.3	123
		Reverse	GAGCGCAGTGCCATCTTAG			
NADH dehydrogenase [ubiquinone] 1 alpha subcomplex assembly factor 3 (*ndufaf3*)	EG940768	Forward	TCCACAGAGGTTCTGGTCCT	0.996	87.8	190
		Reverse	TGCTGATTGGAGGAGGAATC			
peroxiredoxin-like 2a (*prxl2a*); alias *adiporedoxin*	EG907013	Forward	AAACACCACGAGGATGAAGC	0.996	93.0	172
		Reverse	CAATGGGGAGATTTTTGTGG			
prostaglandin D2 synthase (*ptgds*)	EG939741	Forward	TCCACACCATCAAGACCAAA	0.991	98.0	172
		Reverse	GCCTCGGTACATTCACCATT			
transcription factor Sox-9-b (*sox9b*)	XM_014158400	Forward	ACGGACAAAGCGAGTCTGAT	0.981	96.9[h]	102
		Reverse	CCATGCTAGAGGCTGGAGAG			
**Lymphocyte differentiation transcripts**						
interleukin-1 beta (*il1b*)[e]	AY617117	Forward	GTATCCCATCACCCCATCAC	0.997	94.2	119
		Reverse	TTGAGCAGGTCCTTGTCCTT			

interferon regulatory factor 1 (*irf1*)[c]	BT048538	Forward<br>Reverse	GCAATGAAGTAGGCACAGCA<br>CGCAGCTCTATTTCCGTTTC	0.996	91.2	100
dedicator of cytokinesis protein 8 (*dock8*)	DY740796	Forward<br>Reverse	ACTCCCTACTGAGGGGCATC<br>TCCAGCTCTGCTTTGGTTTT	0.997	100.1	157
interferon gamma (*ifng*)[a]	AJ841811	Forward<br>Reverse	CCGTACACCGATTGAGGACT<br>GCGGCATTACTCCATCCTAA	0.994	97.1	133
leukemia inhibitory factor receptor (*lifr*)	EG859398	Forward<br>Reverse	TGGAGCTGATGGAAATAGGG<br>TTTCTCCTGTCCGAATCCTG	0.989	108.8	151
matrix metallopeptidase-19 (*mmp19*)	CK898979	Forward<br>Reverse	GGCCACCCTAGAGATGATGA<br>GATCGCCCTCTTTGTCTCAC	0.994	100.8	167
C-C motif chemokine 4 (*ccl4*)	EG823993	Forward<br>Reverse	AACAATCTGCTTGGGCTACG<br>CTCACCCCTGTCTTGGTGAT	0.995	93.9	174
cyclin-dependent kinase inhibitor 2c (*cdkn2c*)	EG894411	Forward<br>Reverse	CGGAGCAGATGTTAATGCAA<br>GGGTGTCCACATATCCATCC	0.996	99.4	179
kruppel-like factor 4 (*klf4*)	NM_001142713	Forward<br>Reverse	CTACGCACCAGAAGACAGCA<br>TATGGTTTCTCGCCTGTGTG	0.994	103.9	159
C-C chemokine receptor type 5 (*ccr5*)	CK990813	Forward<br>Reverse	TAGGGTGTCTGAGTGCCACA<br>TTGTGGGTTGTCTGAACGAA	0.980	110.2	186
interleukin-7 receptor subunit alpha (*il7r*)	XM_014129609	Forward<br>Reverse	CTCACTGCGGTCAAAGATCA<br>ACTACTCCCAGCCCCAAAGT	0.991	98.1	147
**Lymphocyte function transcripts**						
matrix metallopeptidase-13 (*mmp13*)	CK990871	Forward<br>Reverse	ATTGTTCACGGCTGCTTCTT<br>TTCCAAGTTCGAGGCTTCAC	0.994	107.9	158
dual specificity protein phosphatase 7 (*dusp7*)	CX357422	Forward<br>Reverse	ATCGCACGAGCTTTACGAAT<br>AGTTCGGATGGGTATGTTGC	0.998	99.2	106
E3 ubiquitin-protein ligase RNF144a-a (*rnf144a*)	EG806466	Forward<br>Reverse	ATAGCCTGCTCTGCTGGTGT<br>CAGGGCTAATTCCCATGAAA	0.995	108.1	189
tumor necrosis factor receptor superfamily member 6B (*tnfrsf6b*)[b]	EG881931	Forward<br>Reverse	CCCAGGTCGCACCACTATAC<br>CATCAACTCCCCATCACAGA	0.996	100.1	112
granzyme a precursor (*gzma*)	BT048690	Forward<br>Reverse	TAAAGGTCGCATCCCTCATC<br>CTGCCACAGGGACAGGTAAC	0.997	110.9	131
receptor-interacting serine/threonine-protein kinase 2 (*ripk2*)	DY716681	Forward<br>Reverse	GTGTGAGGGAAAGCAAGGAG<br>CTAGGGCAGGAAGTCTGTGG	0.980	97.1	160
protein kinase c delta type (*prkcd*)	EG930106	Forward<br>Reverse	GGGGGACAACGTGATGTTAG<br>TCAGGAGTCCCACAGAAGGT	0.994	100.2	114
fc receptor-like protein 5 (*fcrl5*)	EG758183	Forward<br>Reverse	GGATGGAGAGTGGTGAGGAA<br>GAGATGGAATGGGAGCATGT	0.993	104.7	190
receptor-type tyrosine-protein phosphatase kappa-like (*ptprk*)	XM_014205048	Forward<br>Reverse	CGCTATGGTCCTGTCCAAGT<br>GAACTGCCTCACCATCAGGT	0.993	83.6[h]	123
t-cell receptor alpha (*tcra*)	BT048618	Forward<br>Reverse	GCCTGGCTACAGATTTCAGC<br>GGCAACCTGGCTGTAGTAGC	0.997	95.3	107
interleukin 13 receptor alpha 1b (*il13ra1b*)	DY708896	Forward<br>Reverse	CGCTGCTGGGTTTACATCTT<br>CTGCGCCACATGACTTCTTA	0.994	100.5	160
**Antigen presenting cell transcripts**						
high affinity immunoglobulin gamma Fc receptor I (*fcgr1*)	EG943149	Forward<br>Reverse	TGAGCCTCCTTGTTGTTTCA<br>GGAACACTGCACTTCAGACG	0.997	102.2[h]	148
tumor necrosis factor ligand superfamily member 14 (*tnfsf14*)	CB513825	Forward<br>Reverse	TGTCATCCAGAAGGAAGGCTA<br>TTTCCCAGACTTGGGGTAAT	0.981	109.0	160
major histocompatibility class I (*mh1*)[a]	AF504022	Forward<br>Reverse	CATGAAGATGTGGAGCATGG<br>AGACCCGTGACTTGAACCAC	0.996	92.4	131
B-cadherin-like (*cdh1*)	GO054831	Forward<br>Reverse	GGCTTGTTCTGAGCTAGTCTGG<br>TTGCTGGTCATGTTCCTGAG	0.996	93.3	131
leucine-rich repeat transmembrane protein FLRT3 (*flrt3*)	EG915183	Forward<br>Reverse	ATCGTACAGGGCGAGAAGAA<br>TGGTGGGTTTGTAAGCCTTC	0.993	100.3	166
n-myc downstream-regulated gene (*ndrg2*)	GE791909	Forward<br>Reverse	CGTACTGTCATCGGAGTTGG<br>TCTGCTCTGTGAGGGAGGAT	0.990	108.9[h]	178
**Transcripts with other functions**						
receptor-transporting protein 2 (*rtp2*)	DW538275	Forward<br>Reverse	GGTTCAGGTGCTCCAAGTGT<br>AAGCATCGCACTTTCACCAT	0.998	98.5	106
receptor-transporting protein 3 (*rtp3*)	EG922647	Forward<br>Reverse	TGCGTCTGTTGGAAGACAAG<br>TTCTTTTCACCTGCGTCCTT	0.995	103.5	181
lipase maturation factor 2 (*lmf2*)	GE795177	Forward<br>Reverse	GTTTGCTTTTGCATCCCTCT<br>GGCATGTATCTGCTCCAACA	0.999	85.5	124
MAP3K12-binding inhibitory protein 1 (*mbip*)	DY697545	Forward<br>Reverse	GAGCTGAAATTGGGTGAAGC<br>TTGGAGATGTGTCGCTGAAG	0.993	109.1	116
guanine deaminase (*gda*)	GE778220	Forward<br>Reverse	GAACGACTACGGCCTGCTAC<br>GTTTCCCCTTCGGACTCTTC	0.993	97.2	169
down syndrome cell adhesion molecule (*dscam*)	GO063234	Forward<br>Reverse	CAGAGTCCCCGAAGGACATA<br>GGTCATCTTGTTGCCATCCT	0.998	101.0	151

tropomodulin-4-like (*tmod4*)[a]	DW569648	Forward	GCTACCACTTCACCCAGCAG	0.998	96.9	153
		Reverse	CAAGGGGCTGAGTTGATTGT			
inactive carboxypeptidase-like protein X2 (*cpxm2*)	CK884675	Forward	AGAGTTCCGGTGATGACGAC	0.995	103.2	166
		Reverse	TGGAACGTGGACACAAAATC			
**Normalisers**						
elongation factor 1 alpha-2 (*ef1a2*)[d]	BT058669	Forward	GCACAGTAACACCGAAACGA	0.997	93.2	132
		Reverse	ATGCCTCCGCACTTGTAGAT			
polyadenylate-binding protein 1 (*pabpc*)[c]	EG908498	Forward	TGACCGTCTCGGGTTTTTAG	0.997	97.5	108
		Reverse	CCAAGGTGGATGAAGCTGTT			
**Infection level TaqMan assay**						
elongation factor 1 alpha-1 (*ef1a1*)[f]	AF321836	Forward	CCCCTCCAGGACGTTTACAAA	0.999	94.6	57
		Reverse	CACACGGCCCACAGGTACA			
		Probe[g]	ATCGGTGGTATTGGAAC			
R. salmoninarum 16S ribosomal RNA	NR_041773	Forward	CAAGGCTTGACATGGATTAGAAAA	0.998	93.2	65
		Reverse	CACCTGTGAACCAACCCAAAA			
		Probe[g]	TGCAGAAATGTACTCCC			

[a] These primer sets were used in Eslamloo et al., (28).
[b] These primer sets were used in Eslamloo et al., (17).
[c] These primer sets were used in Caballero-Solares et al., (44).
[d] These primer sets were used in Katan et al., (43).
[e] These primer sets were used in Zanuzzo et al., (45).
[f] These primer sets were used in Lockhart et al., (36).
[g] TaqMan probe sequence is FAM-5' to 3'-MGB.
[h] The amplification efficiencies of these primers were determined using 4-point serial dilutions of cDNA.
